# Impact assessment of climate policy on Poland's power sector

**DOI:** 10.1007/s11027-018-9786-z

**Published:** 2018-02-12

**Authors:** Tadeusz Skoczkowski, Sławomir Bielecki, Arkadiusz Węglarz, Magdalena Włodarczak, Piotr Gutowski

**Affiliations:** 1 Faculty of Power and Aeronautical Engineering, Institute of Heat Engineering, Division of Rational Use of Energy, Warsaw University of Technology, Nowowiejska 21/25, 00-665 Warsaw, Poland; 2 Faculty of Civil Engineering, Warsaw University of Technology, Warsaw, Poland; 3 Warsaw University of Technology, Warsaw, Poland; 4 Polish National Energy Conservation Agency, Warsaw, Poland

**Keywords:** Poland’s energy sector, EU ETS impact, Energy transformation, Carbon lock-in

## Abstract

This article addresses the impact of the European Union Emissions Trading System (EU ETS) on Poland’s conventional energy sector in 2008–2020 and further till 2050. Poland is a country with over 80% dependence on coal in the power sector being under political pressure of the European Union’s (EU) ambitious climate policy. The impact of the increase of the European Emission Allowance (EUA) price on fossil fuel power sector has been modelled for different scenarios. The innovation of this article consists in proposing a methodology of estimation actual costs and benefits of power stations in a country with a heavily coal-dependent power sector in the process of transition to a low-carbon economy. Strong political and economic interdependence of coal and power sector has been demonstrated as well as the impact caused by the EU ETS participation in different technology groups of power plants. It has been shown that gas-fuelled combined heat and power units are less vulnerable to the EU ETS-related costs, whereas the hard coal-fired plants may lose their profitability soon after 2020. Lignite power plants, despite their high emissivity, may longer remain in operation owing to low operational costs. Additionally, the results of long-term, up to 2050, modelling of Poland’s energy sector supported an unavoidable need of deep decarbonisation of the power sector to meet the post-Paris climate objectives. It has been concluded that investing in coal-based power capacity may lead to a carbon lock-in of the power sector. Finally, the overall costs of such a transformation have been discussed and confronted with the financial support offered by the EU. The whole consideration has been made in a wide context of changes ongoing globally in energy markets and compared with some other countries seeking transformation paths from coal. Poland’s case can serve as a lesson for all countries trying to reduce coal dependence in power generation. Reforms in the energy sector shall from the very beginning be an essential part of a sustainable transition of the whole nation’s economy. They must scale the power capacity to the future demand avoiding stranded costs. The reforms must be wide-ranging, based on a wide political consensus and not biased against the coal sector. Future energy mix and corresponding technologies shall be carefully designed, matched and should remain stable in the long-term perspective. Coal-based power capacity being near the end of its lifetime provides an economically viable option to commence a fuel switch and the following technology replacement. Real benefits and costs of the energy transition shall be fairly allocated to all stakeholders and communicated to the society. The social costs and implications in coal-dependent regions may be high, especially in the short-term perspective, but then the transformation will bring profits to the whole society.

## Introduction

Well-functioning energy sector is a key element of the country’s economic development, stability, wealth and energy security; therefore, its development should be planned with great thoroughness. Climate issues have for long exerted impact on energy policy (IPCC 2014; OECD/IEA 2015). Thus, taking into account the aspects of climate change into national energy policies seems to be an obvious and rational obligation of politicians and energy planners. Being for long the concern to the limited number of the countries committed to the United Nations Framework Convention on Climate Change Kyoto’s Protocol, the issue of addressing the aspects of climate change in energy policy has at last gained a truly global dimension after the 21st Conference of the Parties Paris Agreement. This conclusion is likely to remain valid despite a new, not yet fully recognisable, situation which emerged after the United States (US) declaration to withdraw from the Paris Agreement and which was counteracted by strong re-commitments from other main players including China, India, Japan, Canada, Russia and the European Union (EU).

EU has plans to reduce its greenhouse gas (GHG) emission by 20% in 2020, by 40% in 2030 and then even further by 80…95% in 2050 against 1990 levels. The share of the power sector in these plans amounts to 57…65% in 2030 and 96…99% in 2050 (EC 2011). Further plans set for 2030 aim at least a 27% share of energy from renewable energy sources (RES), 27% energy savings compared with the business-as-usual scenario (BAU). Nowadays, the main policy instrument used by the European Commission (EC) in its efforts to reduce GHG emission in the power sector is its obligatory participation in the EU Emissions Trading System—EU ETS (Directive EU ETS 2009).

Poland has, as a signatory to the Kyoto Protocol since 2002, two legally binding obligations on the GHG reduction, namely, the first one stemming from the protocol to reduce its emissions by 6% in 2008–2012 in relation to 1998 as the base year, and the second one of reduction 20% in 2013–2020 as the EU Member State (MS). Despite successes in meeting these GHG reduction goals, Poland’s climate policy has been heavily criticised by environmentalists who blame insufficient measures undertaken to curb GHG emission caused by extensive coal use (Climate Scorecard 2016; EurActiv 2014). In the opinion of the European Environmental Agency (EEA), *Poland remains one of the most material- and energy-consuming economies of the EU in terms of efficiency* (EEA 2015). As well, Poland is known as a major opponent to the EU climate policy (e.g. O’Rourke-Potocki 2016; Reuters 2017). Adoption to the EU climate policy by Poland may have pivotal importance for the whole EU climate policy which can be blocked or at least effectively obstructed by an MS not fully accepting the EU abatement policy based on the EU ETS framework. This approach is mostly caused by partially understandable fear that adapting domestic energy sector to the climate requirements will cause unbearable burden to the whole economy (see Section 6) and may lead to social unrest in coal regions. The leading role of climate policy in decarbonisation of the whole economy is recognised and acknowledged by the government though even official reports conclude that Poland is not yet prepared to meet the requirements of the EU climate-energy policy (NIK 2016). Obligations imposed on the power sector due to its binding participation in the EU ETS are commonly regarded a real threat looming over the energy future of Poland. Paradoxically, no reliable estimations of the cost of the power sector transformation are available. In the years to come, due to the EU climate policy, especially after the Paris Agreement, it is obvious that the Polish energy mix and energy technologies will have to change. Therefore, a quantitative analysis how the EU ETS affected the conventional power plants in Poland should be of value.

This article addresses in a broad political and economic context a development of coal technologies in Poland’s power sector in the European decarbonisation trend. To this end, a research was made on an economic position of the conventional power plants in 2008–2020 including EU ETS-related costs. This assessment was supported by a 2050 energy demand forecast to check whether current investments in coal-based capacity may lead Poland to commit its GHG emission reduction goal.

The innovation of that article consists in proposing methodology of estimation actual costs and benefits of power stations in a country with a heavily coal-dependent power sector in the transition to low-carbon economy. The article gives answers to the following research questions—what is the cost of participation of currently existing conventional power sector in Poland in the EU ETS over the period 2008–2020? Whether and when will the participation in the EU ETS cause any real threat to the existing coal-fired power plants? May Poland meet the EU emission reduction target for 2050 without a huge reduction of coal usage in the power sector? What should be the recommended generation technologies to replace coal-based plants?

There is a vast amount of literature on the impact of the EU ETS on the European power sector. The authors investigate such mainstream topics as economic effectiveness of the EU ETS in GHG emission abatement; its ability to cause technological transformation to low-carbon technologies; European Union Allowance (EUA) price paths required to meet the climate objectives; extent to which EU ETS costs are passed through to energy prices; and methods of EUA allocation and their impact on the system functioning. Another mainstream research is modelling of different scenarios up to 2050 to see the impact of numerous energy mixes on GHG emissions. Some of these issues, if relevant, will be discussed in subsequent sections. A truly comprehensive review of EU ETS functioning can be found in Healy et al. (2015).

## Methodology

Two models were employed to predict a future perspective of coal-based technologies in Poland’s power system. Namely, the short term, till 2020, based on financial results of existing power plants made available by the Energy Market Agency (ARE S.A.)—energy statistics agency in Poland—and the long-term energy outlook reaching 2050. The emphasis was put on the former whereas the latter was only used to support the conclusions previously obtained. The short-term calculations were made on the basis of commonly accepted economic and emission factors to assess the financial standing of power plants and estimate the costs caused by EU ETS participation (see Section 3.2). The approach adopted is a bottom-up tracking exercise. To end this, the year 2012 of commencing the third trade period in the EU ETS has been chosen as a reference year. The analysis has been limited to 2020 when the fourth period of the EU ETS begins and radically new rules concerning the power sector would come into force. Such a short time perspective expresses the short sight of the EU ETS periods which is one of the main disadvantages of the system. Corporate financial reports of the selected companies active in the power sector in 2014 were analysed. For each of the power stations falling into the four categories adopted, different economic and emission metrics were calculated for the time period of 2008–2014 for which data were available. Basic financial data were extrapolated in the period 2015–2020 or where appropriate kept constant for that relatively short period. Costs directly related to EU ETS were identified to assess their impact on financial standing. Future free allocation of EUA in the power sector was predicted. It was assumed that the energy market prices in 2015–2020 would be not influenced by launching new power plants. Similarly, the RES development was neglected. Since financial data considered as market sensitive, and therefore not publicly available, were used, the results shall then be aggregated within the technology groups to disable back tracing. The methodology applied embraces three main steps—forecasting of most reliable EUA price paths up till 2020, suitable aggregation of power plants into groups, and setting several metrics to measure financial impact of the EU ETS on the power sector (Włodarczak 2016). In the long-term, till 2050, modelling, the Energy Policy Simulator package was employed (see Section 5).

## Challenges of the power sector in Poland

### Dependence on coal

**Fig. 1:**
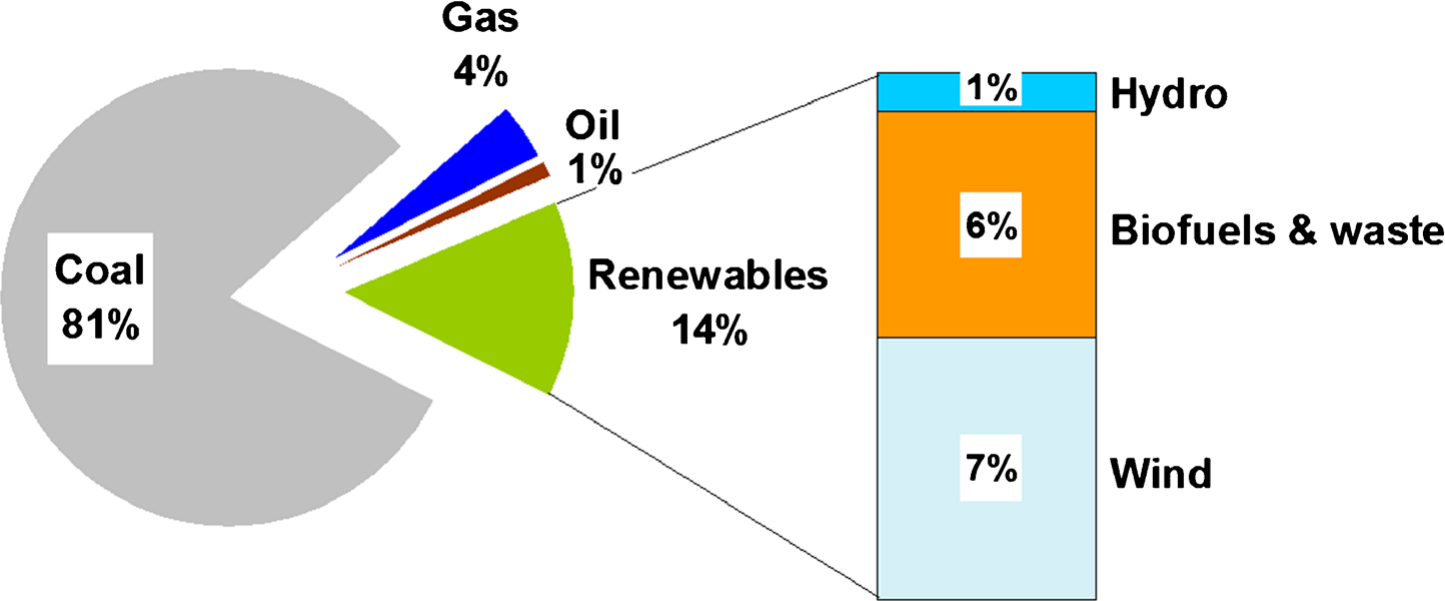
Electrical energy production in Poland in 2014 by the type of fuel. Source: IEA—International Energy Agency (2016a)

Poland’s economy is among the least emissions efficient in the EU—with CO_2_ intensity of gross domestic product (GDP) remaining high in the EU—583 t CO_2_/M€’10 in 2013 with the EU average almost three times lower—198 t CO_2_/M€’10 (World Bank 2014a). The power sector, responsible for around 50% of Poland’s GHG emissions, has ever been indispensably coupled with domestic coal sector. The share of coal (hard coal and lignite) was 52% of total primary energy supply in Poland in 2014 compared to 17% in 28 Member States of the European Union (EU-28) (IEA 2015b). In electricity generation, it amounted to 81% in 2014 (Fig. 1) compared to the EU average of 26% (IEA 2016b). The share of coal in the total electricity production is more than two times higher than in the old 15 MSs (EU-15) average, the highest of all the International Energy Agency (IEA) countries and even higher than in China (Energy Post 2013).

The total installed capacity in the public power sector of Poland was stable 32.3 GW in 2013, 33.6 GW in 2014 and 31.9 GW in 2015, of which the share of combustible fuels was 30.1, 29.3 and 29.6 GW, respectively. The gas power plant capacity was about 1 GW (Polish TSO 2016). Total gross electricity generation in Poland (from all sources) was rather constant in recent years—162 TWh in 2012, 165 TWh in 2013 and 159 TWh in 2014. The share of electricity production is 0.7% of the world’s production which gives Poland the 24th place (2013). Similar statistics concerning Poland in Europe gives 3.4% and the eighth place (BP 2015). Poland’s power sector has always played a key role in the national economy with the share of gross value added of industry of 12.4% in 2005 and 13.4% in 2015. Although, it has now faced several problems, e.g. high reliance of coal, high GHG emissivity, ageing energy infrastructure, underinvestment, lack of coherent long-term development strategy and the requirements to meet environmental standards getting more stringent. However, one of the main threats to the power sector is its inability to remain competitive in the internal European electricity market partly due to high costs of meeting GHG reduction objectives.

Energy sector reforms play a crucial role in the transition to a competitive market economy. It was the case of Poland which took the path from the central governed economy to the market-based economy in the 1990s. The energy sector in Poland has been a subject of an intensive market transformation before the Poland’s EU accession in 2004 and now is mainly driven by the EU climate-energy policy aiming at *clean*, *secure and affordable* energy future (Eur-Lex 2014). At national level, the development of energy sector in Poland has ever been predominantly determined by two main concerns—energy security based on domestic coal deposits and political wish to keep energy prices low. Restructuring of the energy sector in Poland has been a concern of the main international financial organisations, e.g. the International Monetary Fund and the World Bank, which actively supported reforms in energy sectors in many countries in transition (World Bank 2013, World Bank 2014b, 2017a). In a recent opinion of the World Bank, *Poland’s economic ascent is remarkable, but further reforms are needed to meet citizen’s expectations for faster convergence with the European Union* (World Bank 2017a)*.*

Reduction of carbon dependence and meeting the EU climate objectives conflict with exceptionally high reliance of power sector on coal. Reforms in the coal sector in Poland were considered a prerequisite to successful economic transition—Poland made the first ever attempts to reduce its oversized and economically inefficient coal industry. Despite several achievements, the coal sector has never finished a deep reform scaling down production to long-term energy demand taking into account cost-effectiveness of preserving coal as main energy carrier in the power sector. The diminishing role of the coal sector in the national economy is illustrated by lowering its gross value added of industry from 6.9% in 2005 to 3.2% in 2015. The unwillingness to carry out the deep reform in the coal sector, being more of a societal nature and strongly supported by political declarations calling for a restoration or even a development of the current coal mining capacity, has a direct negative impact on the efforts to initiate transformation in the power sector. Politically, the leading role of coal is set and is not a subject of an open public debate. Such an approach disables any knowledge-based governance and pushes decisions in energy sector to undefined future. However, there are many voices from the economic circles calling for a rational vision of diversification of the Polish energy mix, establishing a compromise between low-emission sources and the future role of coal (Energy Forum 2016). More information on Poland’s economy can be found at the World Bank site (World Bank 2014a). Some aspects of energy sustainability are presented in the Global Tracking Framework 2017—Progress Toward Sustainable Energy (World Bank 2017d). A more detailed description on Poland’s energy sector can be found anywhere (e.g. IEA 2015a; Gawlik and Mokrzycki 2016; Nachmany et al. 2015; Ministry of Economy 2015; RWE 2015).

### Participation in the EU ETS

The EU ETS is based on the concept of classical cap and trade system known in economic literature as a cost-effective mechanism to achieve environmental goals by setting a global cap on emissions and establishment of market where rights to emit are traded. The cost of participation in the EU ETS, mainly related to purchasing and selling the EUA, may greatly impact power sector economy, both costs and revenues.

Neuhoff et al. (2006) discussed the importance of emission rights allocation criteria. They remarked that the allowance allocation under the EU ETS differs fundamentally from earlier cap and trade programmes, like sulphur dioxides (SO_2_) and nitrogen oxides (NO_x_) in the USA, and underlined the need for a dynamic change of the rules as the system progresses. Stokka (2015) detailed different methods of allocation and investigated their impact on the Norwegian economy with many references to other countries concluding that the allocation method does not impact operational business decisions at the firm level. Krabbe et al. (2016) report that the new rules adopted for the fourth phase of the EU ETS (2021–2030) may lead to a substantial increase of administrative cost due to continued free allocation).

Till late 2012, the end of the second EU ETS trading period, Poland’s power sector was not heavily troubled by the EU ETS because power installations received all the EUA needed for free. In the third trading period (2013–2020) of the EU ETS, there was a radical change of the EUA allocation rules (Skoczkowski and Wronka 2017). The allocation based on historical emissions (“grandfathering”) was replaced by a more demanding system—a mix of auctioning and output-based allocation (OBA) with some exception left for free allocation. The OBA is based on historical production activity levels multiplied by a product-specific benchmark. Distribution through auctions covered over 40% of the EUA in 2013, and this number will gradually increase—70% by the end of the third phase (2020) and 100% by 2027. Certain installations in other sectors being under the risk of carbon leakage are entitled to get free 100% of the EUA required; manufacturing industry received 80% of its allowances free of charge in 2013, but this number is going to decrease annually until it reaches 30% in 2020. Theoretically, since 2013, the power sector should not receive any free allocations, and in order to fulfil its commitments, it shall purchase all the missing shares at auctions. Although there is a derogation helping EU countries with lower income to mitigate GHG emission from power sector, Article 10c of the EU ETS Directive (Directive EU ETS 2009) allows those MSs to allocate EUA for free to power installations in operation by 31 December 2008 or to installations for which the investment process was physically initiated by the same date, provided that some specific conditions are met. The most important of which is that MSs must invest certain amounts of the revenues from auctioning of the free allocated EUA in the modernisation and diversification of their energy systems leading to GHG reduction. Thus, Poland together with seven other MSs in which in 2006 more than 30% of electrical energy was produced from a single fossil fuel, and GDP per capita at market price did not exceed 50% of the average GDP per capita, is entitled to receive a decreasing number of free EUA to existing power plants for a transitional period until 2019. The situation will further change to become more challenging due to the reforms of the EUA market undertaken by the EC in 2015 (EC 2017) and the new allocation rules to come into force for 2021–2030, and all aimed to restore effective functioning of the EU ETS (European Parliament 2017).

## Short-term impact

The aim of this section is to assess a short-term 2008–2020 impact of the EU ETS on the conventional part of Poland’s power sector.

### Forecasts for EUA prices

**Fig. 2:**
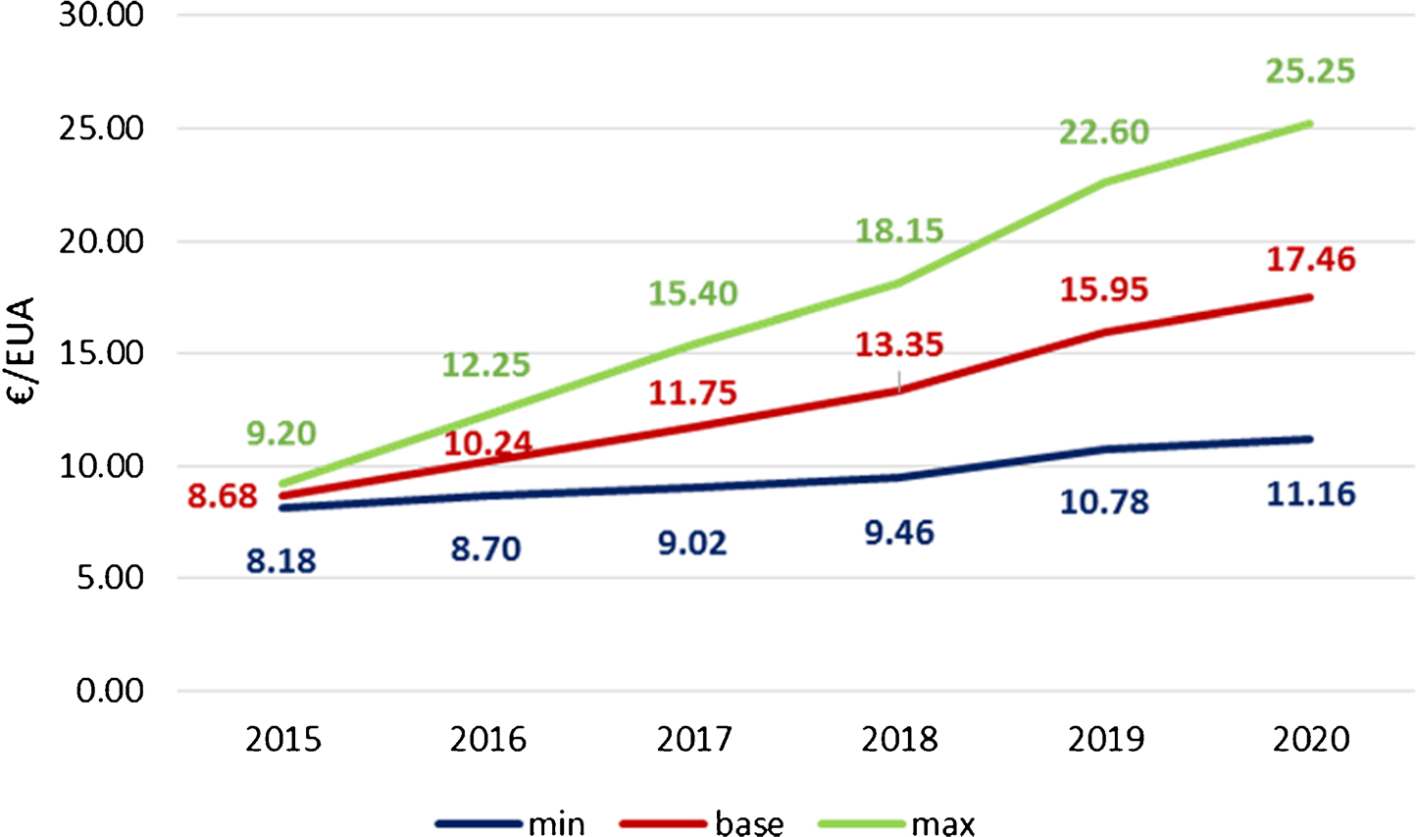
Price scenarios based on forecast EUA price in 2015–2020. Source: own study

Forecasting EUA prices has always posed a problem in the long- or even short-term modelling. Three boundary scenarios of EUA prices were created basing on mid-2015 price projections delivered by Bloomberg New Energy Finance, Commerzbank, Consus, Energy Aspects, ICIS-Tschach, Markedskraft, Nomisma Energia, Point Carbon, Societe Generale, Vertis and Virtuse (Carbon-pulse.com 2015). The base scenario is the average of the published results. The minimum scenario was created as the average of the two lowest scores in the given year, while the maximum scenario as the average of the two highest scores in the given year (Fig. 2).

These price scenarios were compared with the very recent data. The EC predicts EUA price at €10 in 2020 and €35 in 2030. Many MSs use their own projections varying from €6 to €18 per EUA in 2020 and €6…35 in 2030 (EEA 2016a), whereas the EC proposing the Market Stability Reserve (MSR) announced that in the period of 2021–2030, it is aimed to achieve the EUA price of approximately €30, and after 2030, the EC wants to receive an increase of up to €100. In the PRIMES (a model of the energy system used by the EC) modelling, the following path for EUA price increase was used: 10 €’10/t CO_2_ in 2020, 35 €’10/t CO_2_ in 2030 and 100 €’10/t CO_2_ in 2050 (EC 2013). Recently announced (2016) price projections of CO_2_ developed by leading EU ETS analysts forecast a continuous increase of prices from €10/t CO2 up to €22/t CO2 in 2020 (TOE 2016). Seeking reasons for the highly unpredictable EUA prices, it can be noted that the EU ETS has been since 2012 in a permanent state of EUA surplus, principally as a result of a higher than expected reduction of emission since 2008, which was mainly caused by the prevailing economic crisis. However, in-depth research by Koch et al. (2014) revealed that the impact of different factors on allowance price formation from the beginning up to the first year of the third phase of the EU EST is hardly explained by this reason only—there must have been other reasons that led to such a deep fall of the market. Only 10% of price fluctuations can be explained by fundamental market developments (e.g. more extensive introduction of RES than expected, financial crisis, international credits from the Clean Development Mechanism (CDM)). It, therefore, means that a 90% collapse of the price is difficult to explain. Summing up, the EUA price scenarios assumed in this article (Fig. 2) well match all the estimations available now. This approach gave flexible results which are not extreme and even if not realisable, gives good lower and upper bounds. However, since the trajectories chosen are upper the actual price (end 2017), the obtained economic results should be regarded the worst case concerning EUA costs for the power sector.

### Aggregation of power plants

To keep sensitive economic data confidential, all the public power units in Poland were aggregated into four groups according to the used technology and fuel:

1. Combined heat and power plants fuelled with gas (CHP-gas), wherein two units in the group use hard coal in the production process but gas remains the dominant fuel
2. Combined heat and power plants fuelled with hard coal (CHP-coal)
3. Power plants fuelled with hard coal (PP-coal)
4. Power plants fuelled with lignite (PP-lignite)

**Fig. 3:**
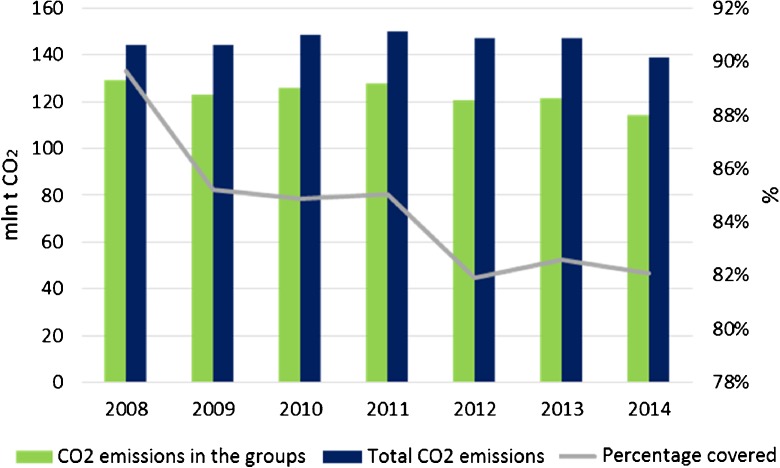
Total emission from the energy sector in Poland compared to emission from the predefined groups of energy plants. Source: own study based on data from ARE S.A. and the Eurostat

This division coincides with the category of the Intergovernmental Panel on Climate Change (IPCC) “1.A.1.a Public Electricity and Heat Production” embracing public thermal power plants, autoproducing combined heat and power (CHP) plants, and excluding marginal contribution of heat plants. The power units considered are all utility power plants operating in Poland in 2014. Industrial and commercial installations were not taken into account due to the lack of data. Only in the case of gas CHP plants, due to a small number of units of that kind and the following risk of violation of statistical confidentiality, this group represents all the gas units in operation in Poland. The total 2008–2014 CO_2_ emission from all the power plants varies between 90 and 82% of the total power sector emission which only confirms the rightness of such partly limited power plant coverage as proposed (Fig. 3).

All essential data were available for the period of 2008–2014 (Table 1), i.e. from the beginning of the second trading phase in the EU ETS until the end of the second year of the third period so that the change of EUA allocation rules from free allocation to auctioning in the third phase was included. Decommissioning of units within each group was taken into account whereas new capacities foreseen till 2020 were not considered since observing actual construction progress their putting into fully commercial operation was very much doubtful. Moreover, the aim of this work was to assess the impact on the existing capacity disregarding possible minor market changes caused by a newly commissioned capacity.

**Table 1:** Aggregated data used for all the groups together with units and shortcuts used thereafter

Data	Shortcut	Unit
Electric power	P_E_	MW
Gross energy production	E_P_	MWh
Net heat production	E_H_	GJ
CO_2_ emission	E_CO2_	t
Revenues generated from sales of electrical energy and heat	R_1_	€
Total costs incurred in the generation of the revenue	C_1_	€
Profit/loss on sales of electrical energy and heat	P/L_1_	€
Other revenues	R_2_	€
With revenues generated from sales of EUAs	${R}_{2_{CO_2}}$	€
Other costs	C_2_	€
With costs incurred in purchase of EUAs	${C}_{2_{CO_2}}$	€
Profit/loss on sales of electrical energy and heat with other revenues and costs considered	P/L_2_	€
Financial revenues	R_3_	€
Financial costs	C_3_	€
Profit/loss on sales of electrical energy and heat with other and financial revenues and costs considered	P/L_3_	€

### Emission and economic factors

The main goal was to determine how the EU ETS impacts the profitability of different units (grouped as above), as well as to calculate the emission factor depending on the fuel they use. To analyse the data thoroughly, the following metrics were used (symbols in the formulas are explained in Table 1).

#### Emission factor (EF1)

1 $$ {EF}_1=\frac{E_{{\mathrm{co}}_2}}{E_{\mathrm{p}}+0.278{E}_{\mathrm{H}}}\kern0.5em \left[\mathrm{t}{\mathrm{CO}}_2/\mathrm{MWh}\right] $$

Formula (1) differs from the methodology provided in the IPCC Guidelines (IPCC 2006). It was decided to include not only the electrical energy production but also heat, because it well suits CHP. The default emission data are given in literature, but the aim here was to obtain actual values for different technologies applied in Poland.

#### Balance of revenues and costs of participation in the EU ETS

2 $$ {B}_{\mathrm{RvsC}}=\frac{R_{2_{{\mathrm{CO}}_2}}-{C}_{2_{{\mathrm{CO}}_2}}}{E_{\mathrm{p}}+0.278{E}_{\mathrm{H}}}\kern0.5em \left[\text{\EUR} /\mathrm{MWh}\right] $$

Balances of revenues and costs were done for all the groups. The ratio of the difference between revenues and costs to the energy production was defined in order to enable the comparison of the performance of each technology and fuel.

#### Profitability with CO_2_ impact included (*P*_CO2 incl_)

3 $$ {P}_{\mathrm{CO}2\mathrm{inclu}}=\frac{P/{L}_3}{R_1+{R}_2+{R}_3}\kern0.5em {\displaystyle \begin{array}{c}\left[\%\right]\end{array}} $$

Profitability was determined by dividing total profit/loss on sales of electrical energy and heat with financial and other revenues and costs considered by the sum of revenues generated by the unit.

#### Profitability with CO2 impact excluded (*P*_CO2 excl_)

4 $$ {P}_{{\mathrm{CO}}_2\mathrm{excl}}=\frac{P/{L}_3+{C}_{2_{{\mathrm{CO}}_2}}-{R}_{2_{{\mathrm{CO}}_2}}}{R_1+{R}_2+{R}_3-{R}_{2_{{\mathrm{CO}}_2}}}=\frac{P/{L}_{3\_{\mathrm{CO}}_2\mathrm{excl}}}{R_1+{R}_2+{R}_3-{R}_{2_{{\mathrm{CO}}_2}}}\kern0.5em \left[\%\right] $$

where *P/L*_3___CO2 excl_ stands for profit/loss on sales of electrical energy and heat with financial and other revenues and costs considered, excluding the impact of the EU ETS.

Profitability without CO_2_ trading was counted in order to show the impact of the EU ETS on the power business.

### Results and discussion

#### CO_2_ emission in the period of 2008–2014

**Fig. 4:**
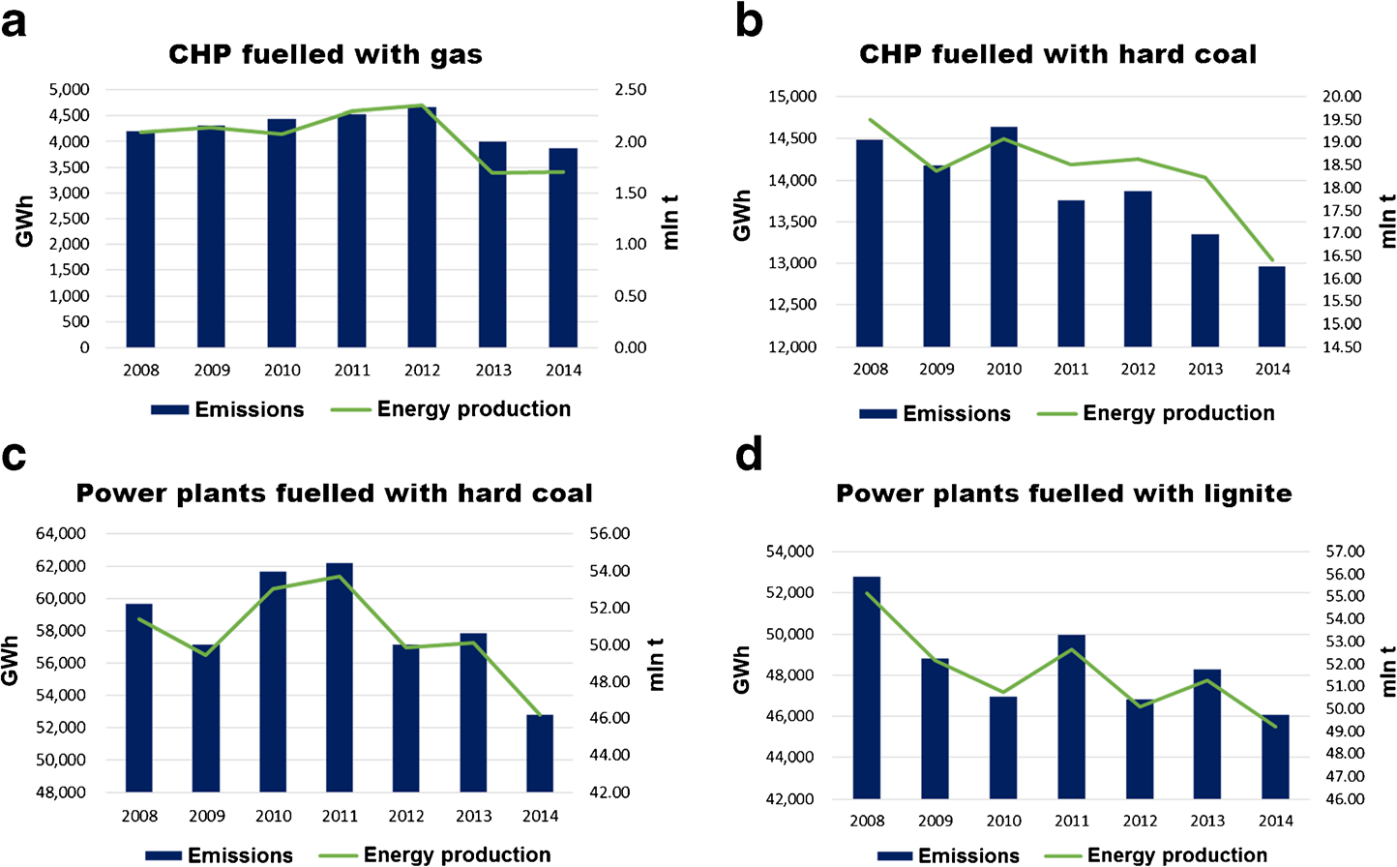
Correlation between energy production and CO_2_ emissions in the different groups of power plants. Source: own study based on data from ARE S.A.

There is a strong correlation between the energy produced and emission in each of the groups—the absolute CO_2_ emission depends on the fuel used and the energy produced (Fig. 4).

The development of high-efficient CHP is one of the pillars of the EU energy efficiency policy (Directive EED 2012). What matters is that using hard coal in CHP plants can cut the emission factor by half in comparison with a power plant producing only electrical energy. It should be considered while exploring new investment opportunities especially in times when the EUA prices are inevitably to rise.

**Fig. 5:**
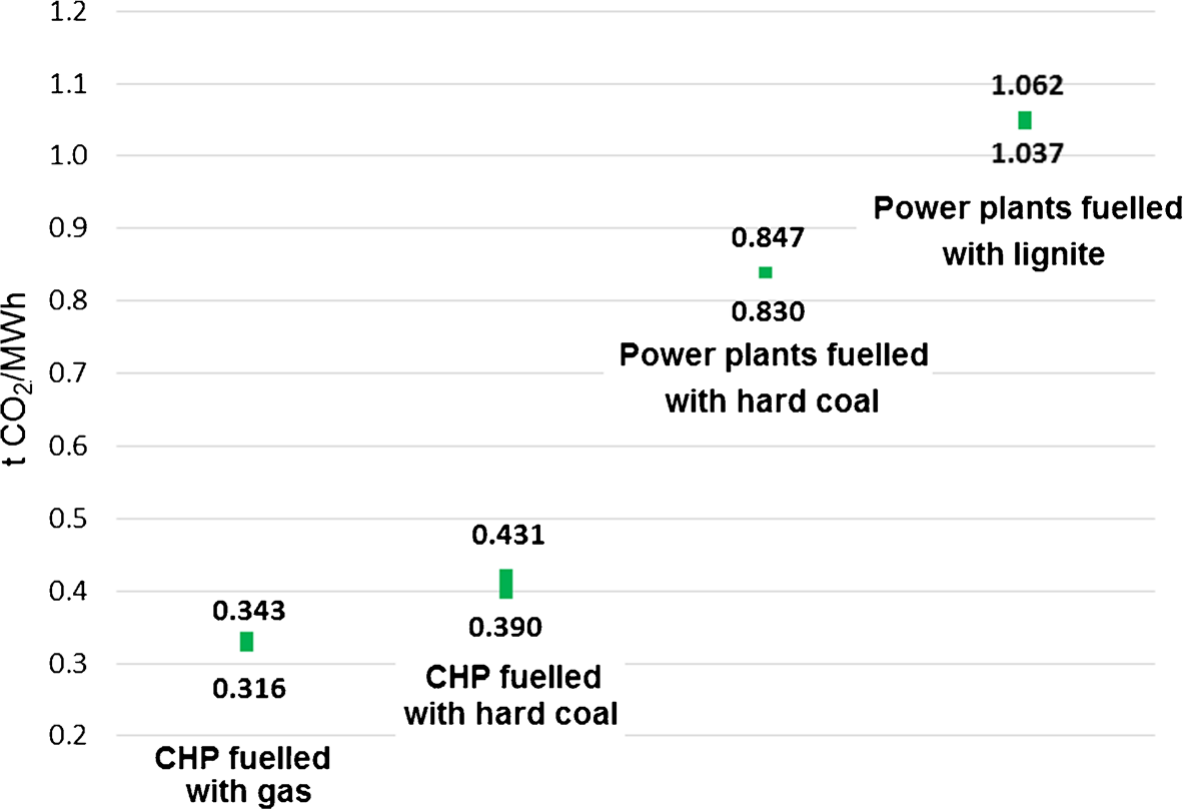
Range of emission factors for the predefined groups in the period of 2008–2014. Source: own study based on data from ARE S.A.

The emission factors reveal substantial, however expected, differences among power technologies used in Poland (Fig. 5). The most emissive fuel is lignite—over 1 t CO_2_/MWh. In contrast, the least emissive are gas-fuelled CHP plants. In Poland, the number of gas-fuelled CHP plants is small due to a high gas/coal price ratio, and they are mostly used as peak capacity. Their environmental benefits as compared to other technologies have not yet been properly valued, partly due to low EAU-related costs. The situation is likely to change since there are several gas-fuelled plants under consideration following the need to use possible oversupply of gas under import contracts. Poland’s national inventory report covering all the EU ETS energy installations gives the average emissivity factor of EU ETS power installations of 0.8 t CO_2_/MWh (KOBIZE 2015). The figure is very close to the results obtained in our analysis; however, our results distinguish between different technologies.

A large improvement in emission intensity from public electricity and heat generation in the EU-28 was observed in 1990–2012; as in 2012, it was 0.566 t CO_2_/MWh compared to 0.754 t CO_2_/MWh in 1990. In 2000–2010, the CO_2_ emissivity fell by 29% (on average 1.7% per year). Since 2010, however, CO_2_ emission intensity has increased by 2.6% per year due to a larger share of coal and lignite temporarily replacing gas. Comparing EU-28 and Poland’s average emission intensities in 2013, one can find 0.558 t CO_2_/MWh and 0.713 t CO_2_/MWh, respectively, which is unexpectedly a good result (1:1.28).

On the list of top 30 emitters in 2015 in the category combustion plants, there are four Polish power plants: Bełchatów with annual emission 37.1 Mt CO_2_ and no change against 2014 (0%), Kozienice 11.4 Mt CO_2_ (+ 4%), Turów 7.6 Mt CO_2_ (− 7%) and Rybnik 6.5 Mt CO_2_ (− 10%) (EEA 2016a). These not improving emission records trigger public and political criticism since the power plants are recipients of free EUA allocations under Article 10c. Especially, the case of the Polish Bełchatów power plant is widely publicised (CEE 2016; EC 2015b)^.^ It is claimed that the funds allocated to the power plant are used for restoring coal capacity instead of reducing GHG emission. There are also threatening voices calling lawmakers for excluding coal power plants from the enabled by the EU ETS reform energy transition funds. In contrast, in the World Bank’s RISE ranking (World Bank 2017c) embracing regulatory indicators for sustainable energy overall score, Poland with 78 points is globally on the 24th position and 14th in the EU (energy access—100, renewable energy—78, energy efficiency—57). However, the ranking overvalues countries with full access to energy and, therefore, is not fully suitable to asses highly developed countries.

In PRIMES model, carbon intensity of power generation from thermal plants steadily decreases: 0.41 t CO_2_/MWh in 2020, 0.33 t CO_2_/MWh in 2030 and 0.16 t CO_2_/MWh in 2050. It is accompanied by a corresponding rise in efficiency of thermal electricity production—38.4% in 2010, 40.8% in 2020, 42.7% in 2030 and 44.6% in 2050. This diminishing trend is not reflected in Poland’s power sector. The average efficiency of Polish thermal power plant was 33.7% in 2011 and 34.2% in 2013 that was below the EU average—38.5 and 36.3% respectively in these years (WEC 2016). Only the new coal units planned for commissioning till 2020 will exceed the current EU average, e.g. in Opole 45.5% net (2 × 900 MW), in Kozienice 45.59% (1075 MW), in Jaworzno 45.9% (910 MW) and in Turów 42% (450 MW). It can be assumed that Poland’s energy sector has now reached stage I of the Clean Coal development, i.e. being retrofitted and with new capacity being in line with current standards of energy efficiency and emission reduction of SO_2_, NO_x_ and dust requirements. Further progress is limited since achieving energy efficiency greater than 50% in coal-fired technologies still requires research especially when it is to be combined with carbon dioxide capture and storage (CCS) i.e. Clean Coal Stage III (Kavouridisa and Koukouzas 2008). Bryant (2016) discussed the impact of high coal dependence in highly concentrated power sector in the EU in 2005–2012 on socio-spatial concentration and centralisation of capital and GHG emission (EC 2015b). In the case of Poland, he concludes that ownership relationship changes supported concentration of GHG emission under the governmental control. The process has recently been even deepened as some of the coal-fired power stations were sold back (indirectly nationalised) by international energy companies like Vattenfall or Électricité de France (EDF) to state-controlled power companies. This makes the government particularly strong and potentially an effective enabler in the GHG emission abatement policy. It also puts extraordinary responsibility on the government.

#### Allocated and verified allowances in the period of 2008–2014

**Fig. 6:**
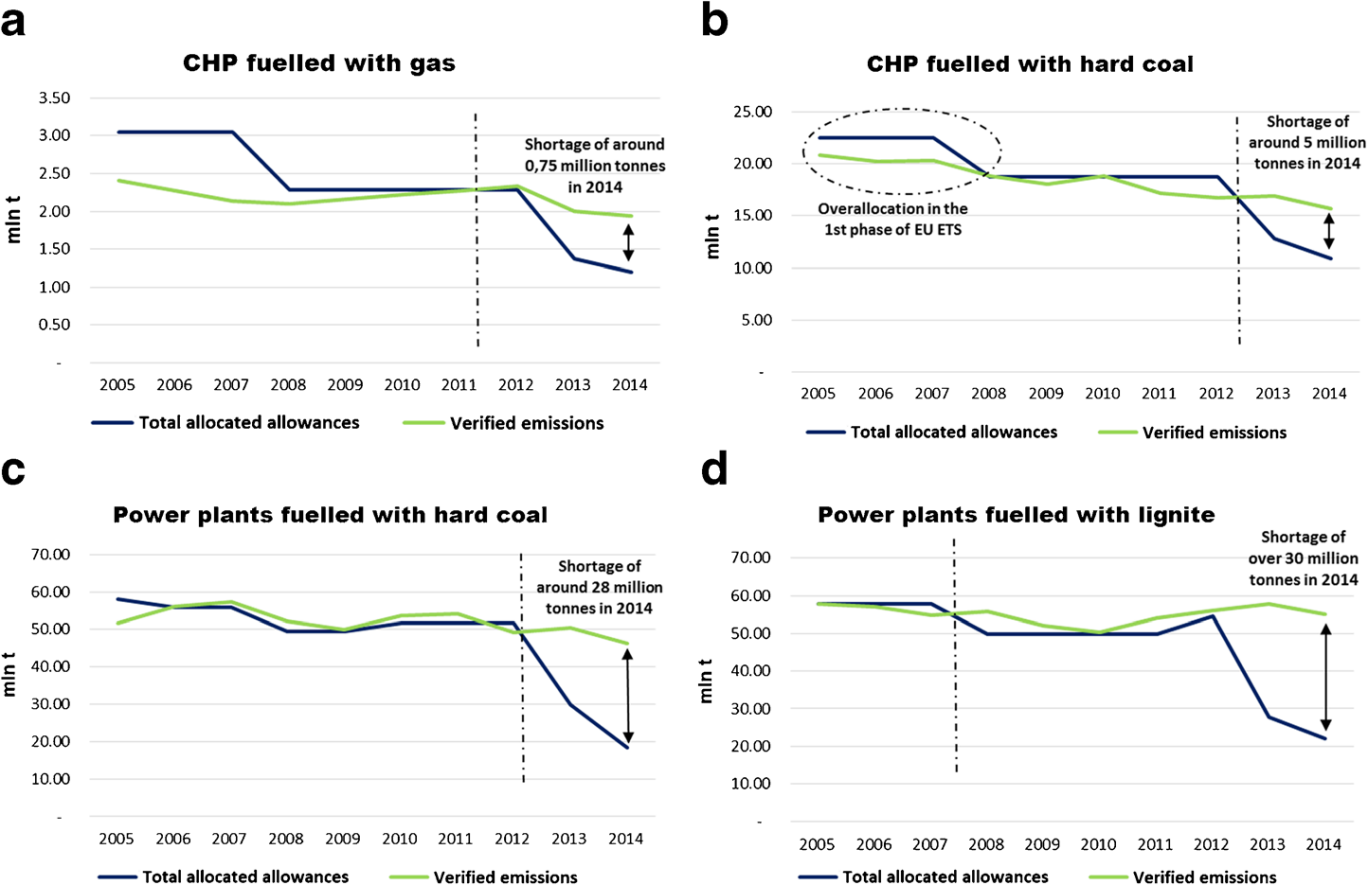
Total freely allocated allowances and verified emissions in the predefined groups. Source: own study based on data from EUTL

Participation in the EU ETS compels installation operators to preserve their emissions at some predefined level. The comparison of the allocated EUA and the emissions verified by the EC over all the EU ETS phases is of interest to see whether the plants were able to make profit by selling over-allocated EUA or they had to buy the missing ones. It was done using data from the EU Transaction Log (EUTL). This database provides all the information on the individual power plants; therefore, it was possible to assign verified individual emissions into the established groups. The results show the extent to which the situation of the plants changed with the commencement of the third trading phase (2013) and the alteration of rules of allocation, e.g. from free allocation to auctioning (Fig. 6).

While in the first trading phase (2005–2007) the majority of the groups had experienced over-allocation which probably resulted in additional revenues, in the subsequent second trading period (2008–2012), the total EUA allocated remained sufficient only in the case of CHP plants fuelled with hard coal and until 2011 in units fuelled with gas. At the same period, EUA shortages experienced by other groups were not substantial. The beginning of the third trading period (2013–2020) changed the situation completely. In all cases, the shortage is getting higher every year, and the next years are rather not likely to bring any relief providing that the freely allocated share is to completely diminish by 2020.

Comparing Poland’s rate of emission from combustion over 2005–2014 period with other three main EU polluters (Germany, the UK, Italy), one can conclude that Poland has stabilised its emission while other countries show a certain degree of decrease (EEA 2016a). It can suggest that despite a growing share of RES in energy mix and efforts to reduce emission from individual power units by improving energy efficiency, a kind of stagnation caused by tapping all low-cost energy saving potential can be observed in Poland’s power sector.

#### Production costs of power plant

**Fig. 7:**
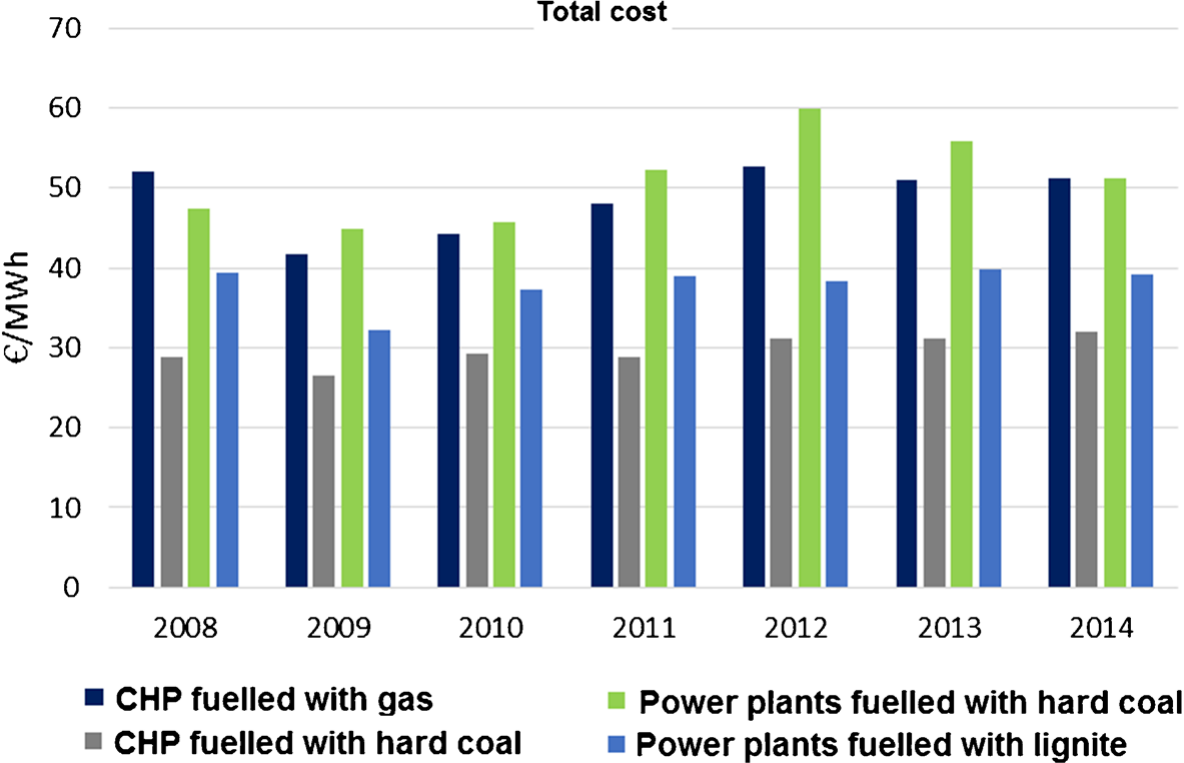
Average total cost of producing 1 MWh in the predefined groups in years 2008–2014. Source: own study based on data from ARE S.A.

Costs of production of electrical energy and/or heat differ with the technology and fuel used. In 2008–2014, the total costs ranged between €60/MWh and €27/MWh. The highest values were for hard coal power plants and gas CHP plants in 2012 (as gas is the most expensive fuel in Poland), and the lowest for hard coal CHP plants in 2009 (Fig. 7). The average production cost of electrical energy in coal-fired power plants was €44/MWh in 2014 and €46/MWh in 2015, i.e. 2.8% higher (URE 2017). Just to compare, the wholesale electrical energy sold on the competitive market of the Polish Power Exchange was lower—€42/MWh in 2014 and €41/MWh in 2015.

#### Impact of the EU ETS costs on energy costs

For the purpose of this study, the total cost and revenues of energy production consist of the following:

- Costs incurred in the generation of the revenue which encompass all the production costs except for CO_2_
- Other costs which embrace CO_2_-related costs (trading, purchase of EUA)
- Financial costs

**Fig. 8:**
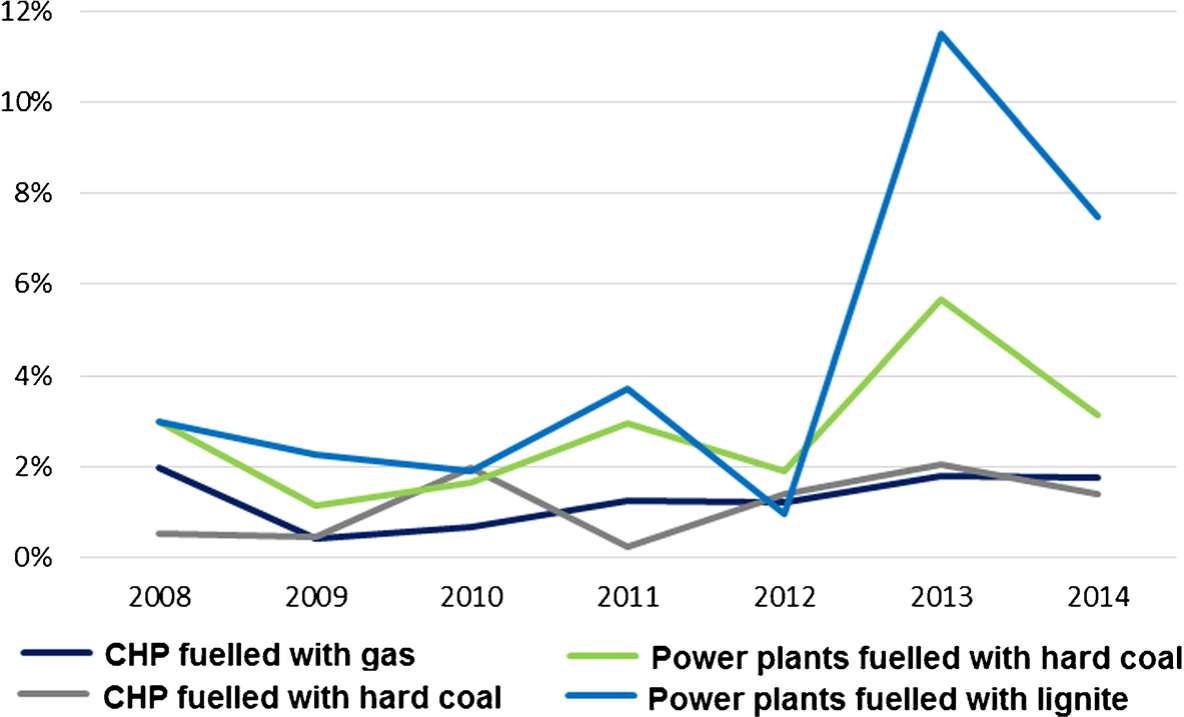
Fracture of CO_2_ costs in total costs of 1 MWh production. Source: own study based on data from ARE S.A.

The costs of participation in the EU ETS categorised in the accountancy of power plants as a part of “other costs” were filtered from the financial data available. They vary between 0.2 and 11.5% of the total costs of production in the assessed groups. The biggest fraction of the CO_2_ costs when compared to other costs is in the case of lignite-powered power plants (up to 11.5% in 2013) and hard coal power plants (up to 5.7% in 2013). They are quite acceptable and stable for CHP plants since they barely exceed 2% (Fig. 8).

#### Balance of revenues and costs

**Fig. 9:**
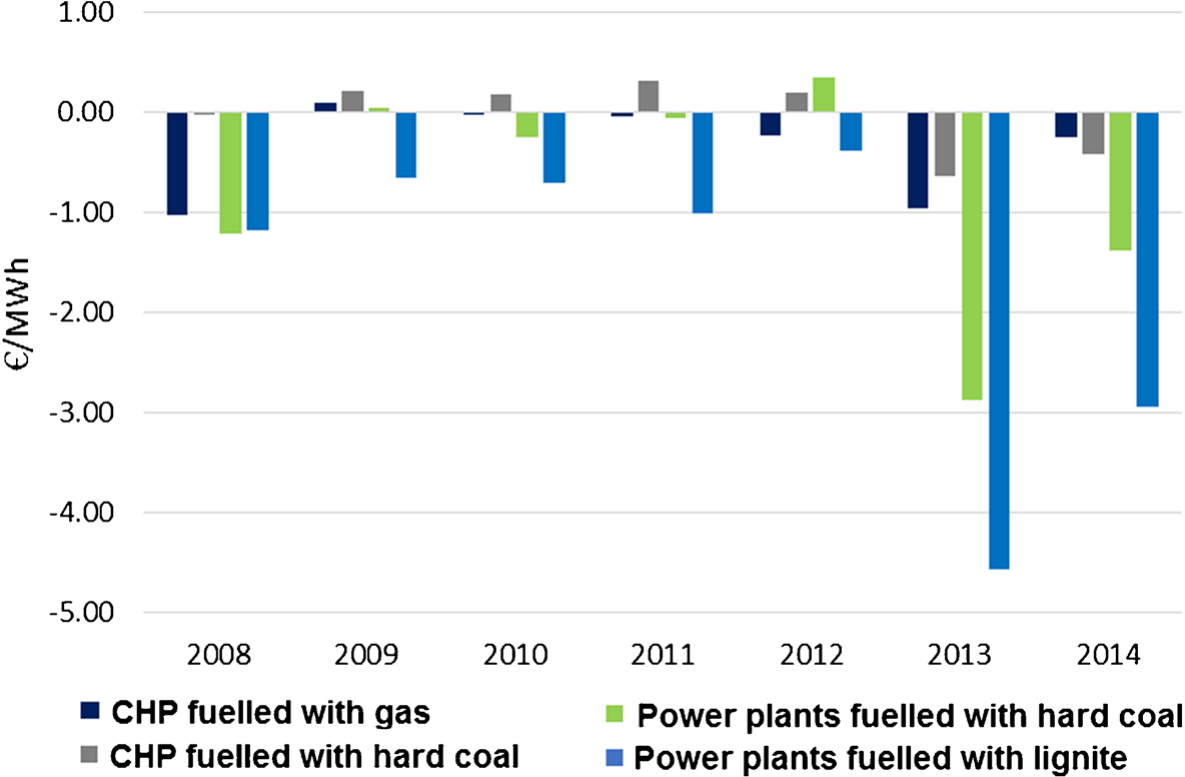
Balance of revenues and costs for 1 MWh due to the EU ETS participation in the period of 2008–2014. Source: own study based on data from ARE S.A.

The costs and revenues due to participation in the EU ETS were counted and expressed for one energy unit (MWh) of electrical energy and heat generated (Fig. 9). As expected, additional costs and revenues are considerably smaller for CHP technology, while the costs borne by the power plants are substantially higher. In the second trading period, some units were able to sell remaining EUA thus generated additional revenue—they benefited from the EU ETS participation, whereas it was never the case for lignite power plants and rare for power plants fuelled with hard coal.

The situation radically changed with the start of the 3rd trading phase as from 2013 only a fracture of EUA remained allocated freely. All shortages had to be covered by EUA purchase at auctions which increased the operational costs. Notably, in 2013 the costs generated by EUA purchase were higher than in 2014 due to a growing oversupply of EUA and the following drop of prices. It might also be a sign that power sector, noticing that the price of allowances started rising in May 2013, decided to take preparatory steps for the decrease of the number of freely allocated allowances and purchased too many EUA. In the extreme case of the hard coal power plants it resulted in €4 /MWh increase of production costs. However, the situation changed after the 3rd trading phase had commenced as in 2013 the period of growing losses began and it is likely to last until 2020 and afterwards. The losses are likely to steadily increase unless the power sector undertakes provisions to reduce its GHG emission.

#### Profitability

**Fig. 10:**
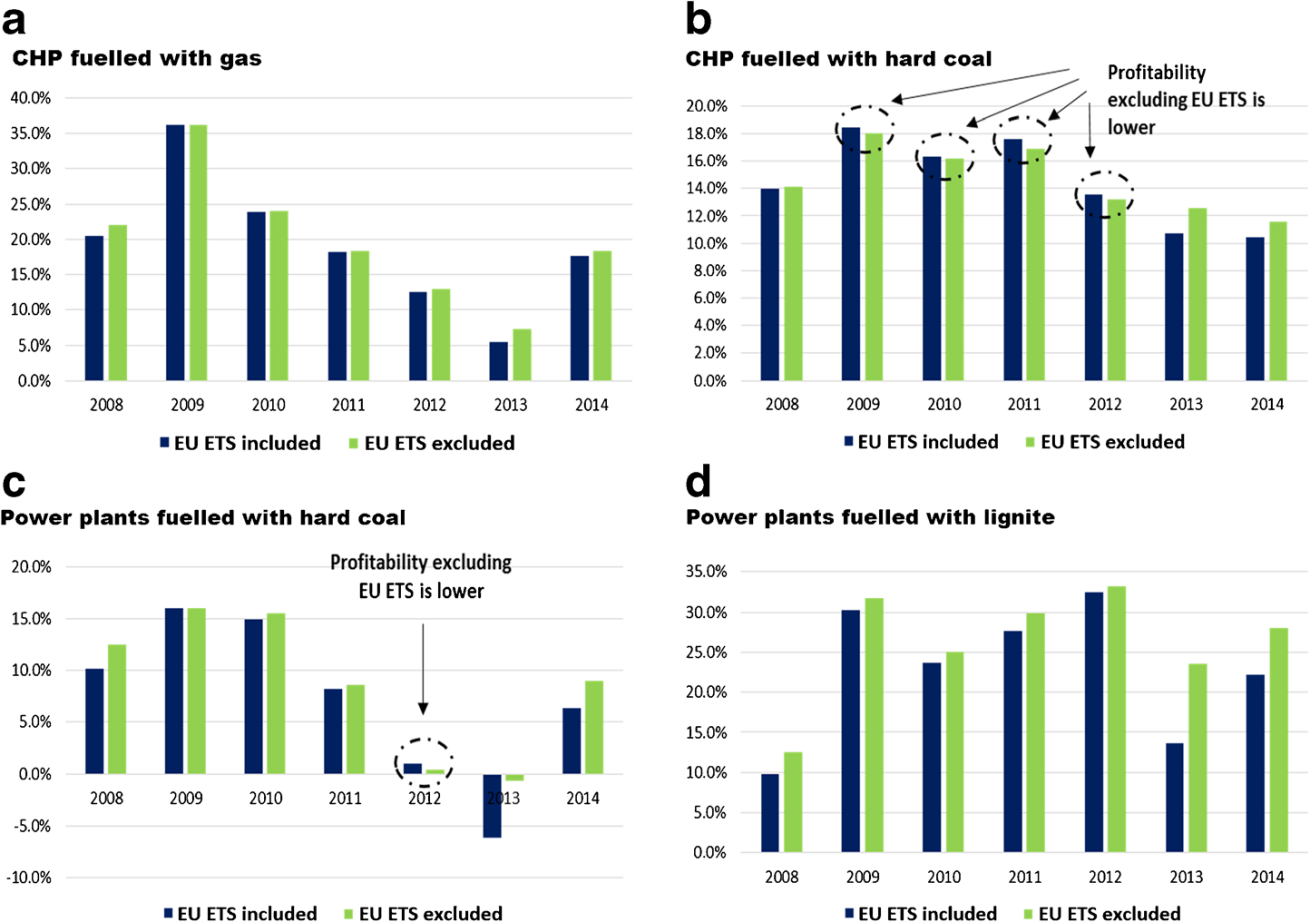
Comparison of profitability in the predefined groups with and without the EU ETS. Source: own study based on data from ARE S.A.

Yearly profitability, measured as a ratio of financial profit (profit/loss count) and total revenues, was calculated for each fuel/technology group (Fig. 10). In the period of 2009–2012 for hard coal CHP plants and in 2012 for hard coal power plants, the profitability with the EU ETS excluded was lower, than that in the non-ETS case. That means that the participation in the system allowed the units (seen as a group) to gain an additional profit. The reason for this situation was that the number of freely allocated EUA that they obtained was bigger than their actual need. In all remaining cases, the profitability was either slightly (CHP-gas) or significantly higher (PP-lignite) with the exclusion of the EU ETS impact.

### Short-term perspective till 2020

Forecasting of future financial standing for the groups till 2020 required to predict future emissions and the EUA needed to cover them, to assess the free allocated EUA and then to estimate the number and costs of the missing EUA.

#### Predicted emissions

**Fig. 11:**
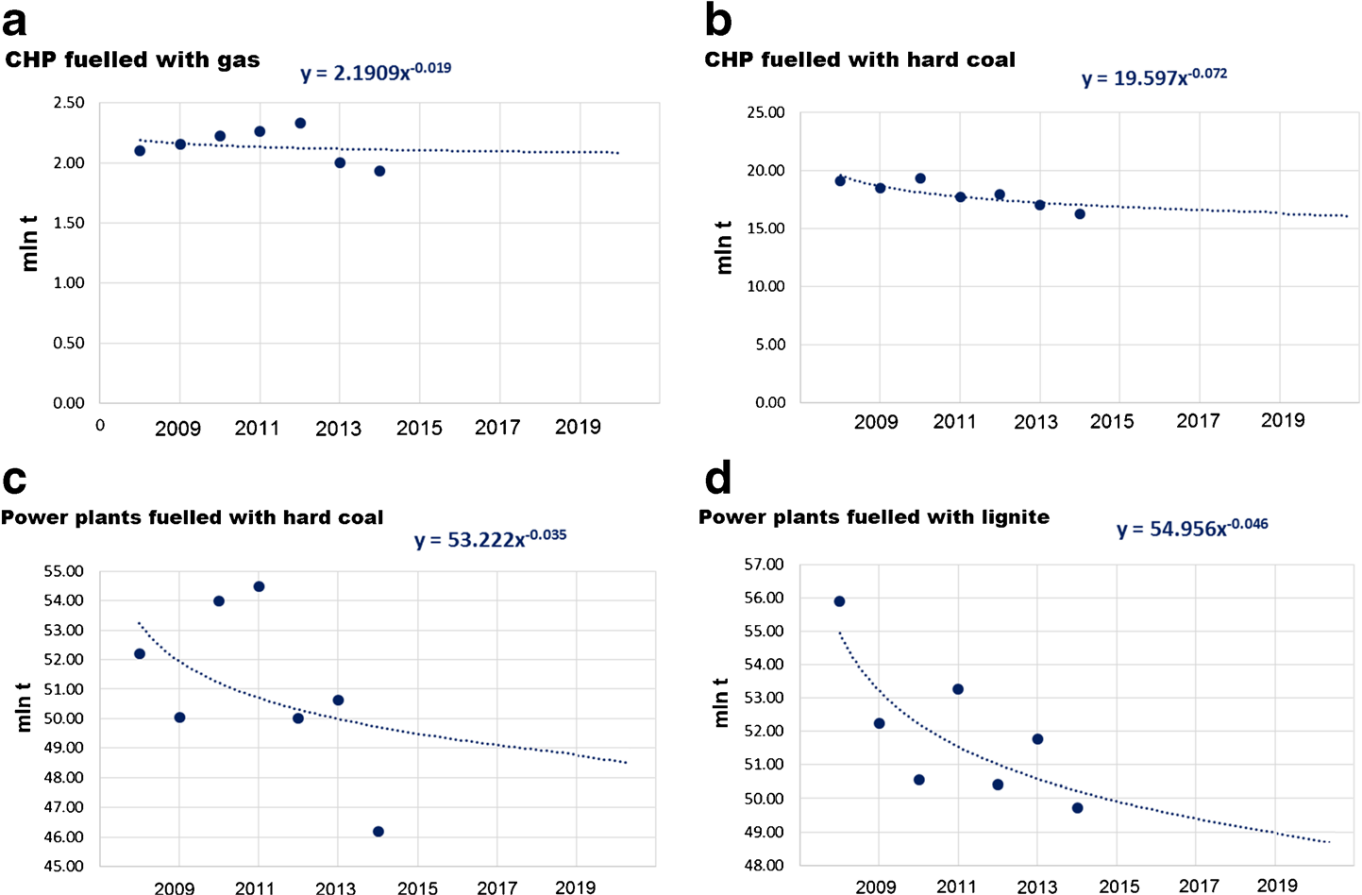
Forecast of future CO_2_ emission in the predefined groups over the period 2008–2020. Historical data used for the forecasting marked with dots. The equations describe a future trend after 2014. Source: own study based on data from ARE S.A.

The power capacity and energy production till 2020 were estimated according to governmental projections (Ministry of Economy 2009; Ministry of Energy 2015). The power trend line was built to predict emission levels in the period of 2015–2020. To estimate the future emissions for the predefined groups, trends based on the data from 2008 to 2014 were used (Fig. 11).

**Fig. 12:**
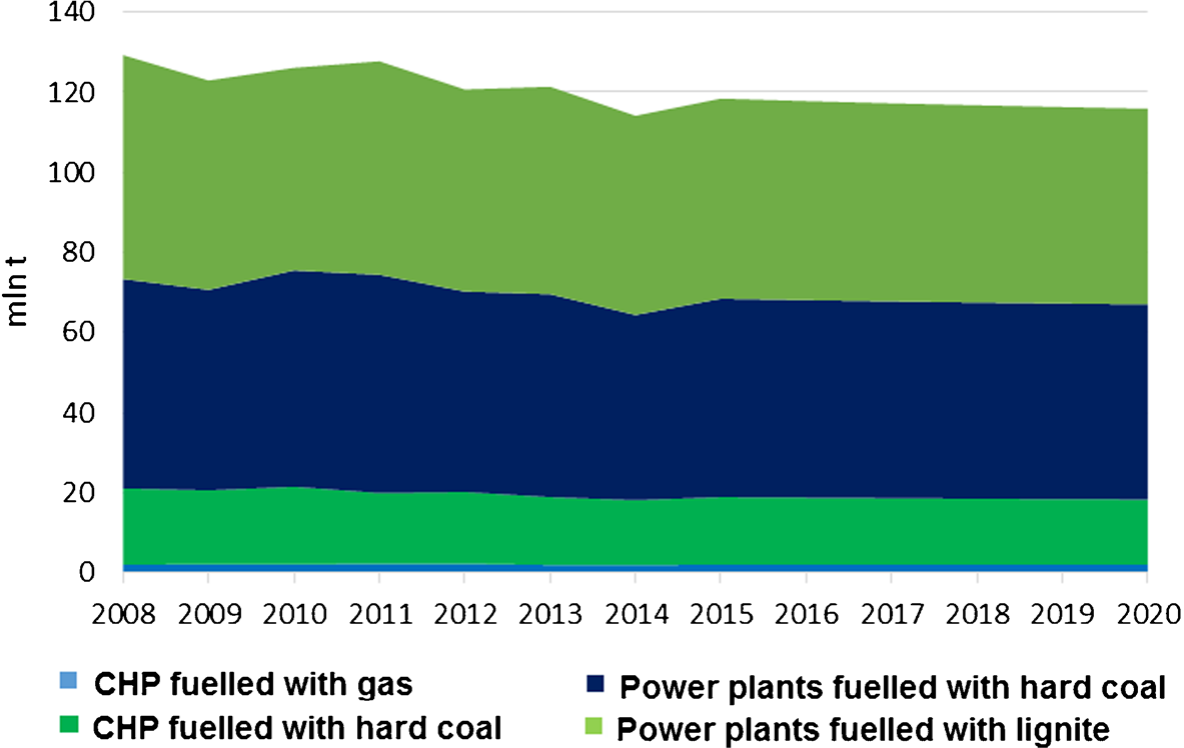
Forecast of future emission from the predefined groups. Source: own study based on data from ARE S.A.

The total emission of the groups will decrease by 13.4 million tonnes of CO_2_ when comparing levels in 2020 and 2008. The decrease in the third trading phase alone will be of 5.5 million tonnes (Fig. 12).

#### Free allocation of the allowance

**Fig. 13:**
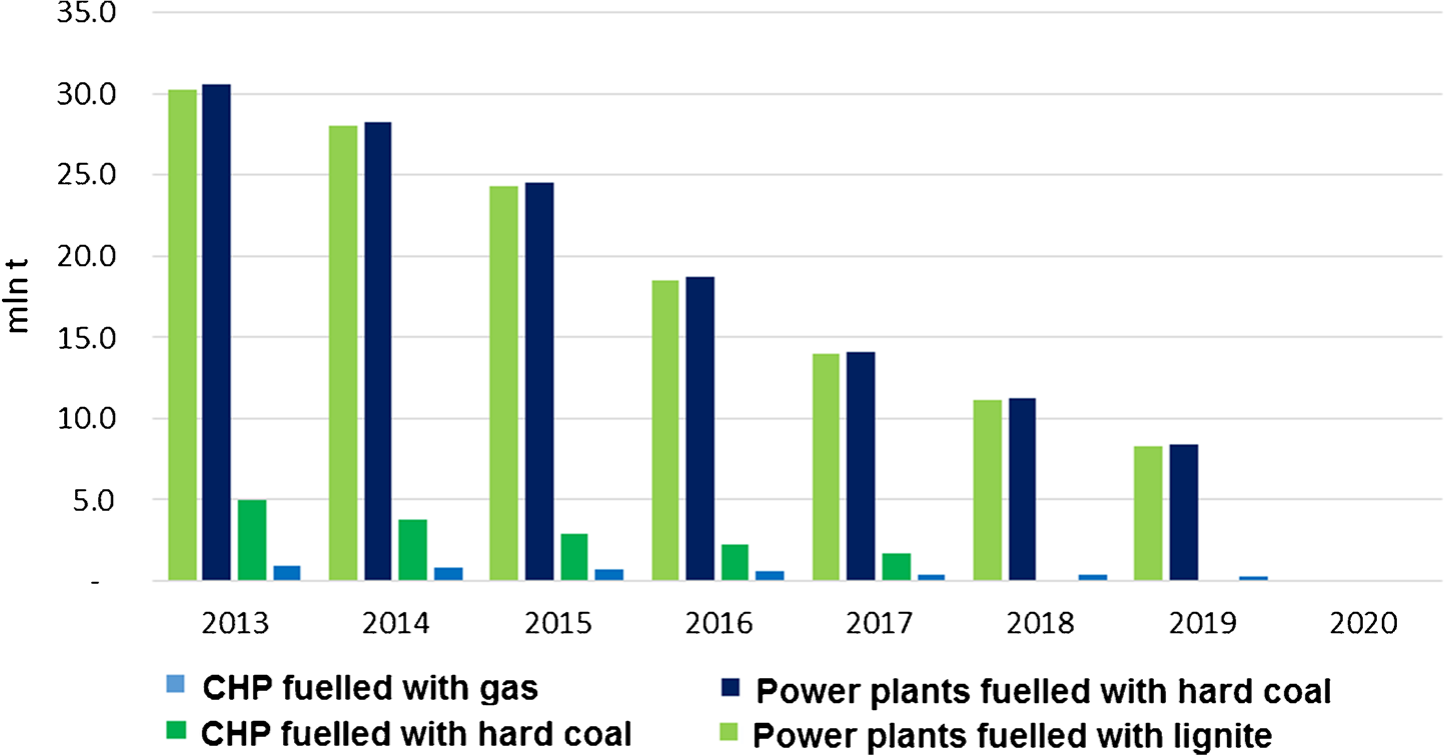
Planned free allocated EUA due to derogation mechanism based on Article 10c of the EU ETS Directive. Source: own study

The free EUA are individually assigned to power units according to governmental regulation (Fig. 13) although each year, the amount is adjusted according to the progress made in compliance with the planned investment. Over the third trading period, Poland is likely to obtain 424 million of free EUA for the whole power sector (Cabinet of Ministers 2014) as a part of the derogation mechanisms.

The future amount of EUA obtained through derogations of Article 10c had to be predicted. Historical data available covered only 2013 and 2014, the period too short to estimate the trend. Moreover, the use of free EUA in 2013 and 2014 was below schedule in all the predefined groups with growing tendency to diverge. Thus, it was assumed that the compliance for the whole period of 2015–2020 in each group separately would be constant and equal the average from the years 2013–2014, i.e. CHP-gas—84%, CHP-coal—86%, PP-coal—80% and PP-lignite—84%. However, later it turned out that the estimation was too optimistic since the actual share of all free allocated EUA used under Article 10c by power sector in 2013–2015 amounted only to 40% which was the lowest score in all eligible MSs (EEA 2016a). Despite the fact, the assumption still seems reasonable since the refurbishment and modernisation of power units require substantial investments which will force companies to struggle to maximise the share of free EUA and to accelerate investments at the end of the eligible period.

#### Costs due to allowance shortage

After forecasting emissions and freely allocated EUA in the years 2015–2020, the shortage of EUA for the groups was estimated. Considering the previously set EUA price scenarios forecasts, the estimation of the costs generated by participation in the system was made. The predicted shortage of allowances was simply determined by subtracting the free allowances to be obtained in the period of 2015–2020 from the forecasted emission levels (Table 2).

**Table 2:** Predicted shortage of EUA in the predefined groups in 2015–2020 (in million EUA)

Year	2015	2016	2017	2018	2019	2020
CHP-gas	1.05	1.27	1.43	1.54	1.65	1.90
CHP-coal	7.53	9.29	10.66	11.59	12.47	14.30
PP-coal	29.35	33.92	37.44	39.62	41.84	48.46
PP-lignite	29.25	33.90	37.48	39.68	41.91	48.72
Total	67.18	78.38	87.01	92.43	97.87	113.38

Source: own study

**Fig. 14:**
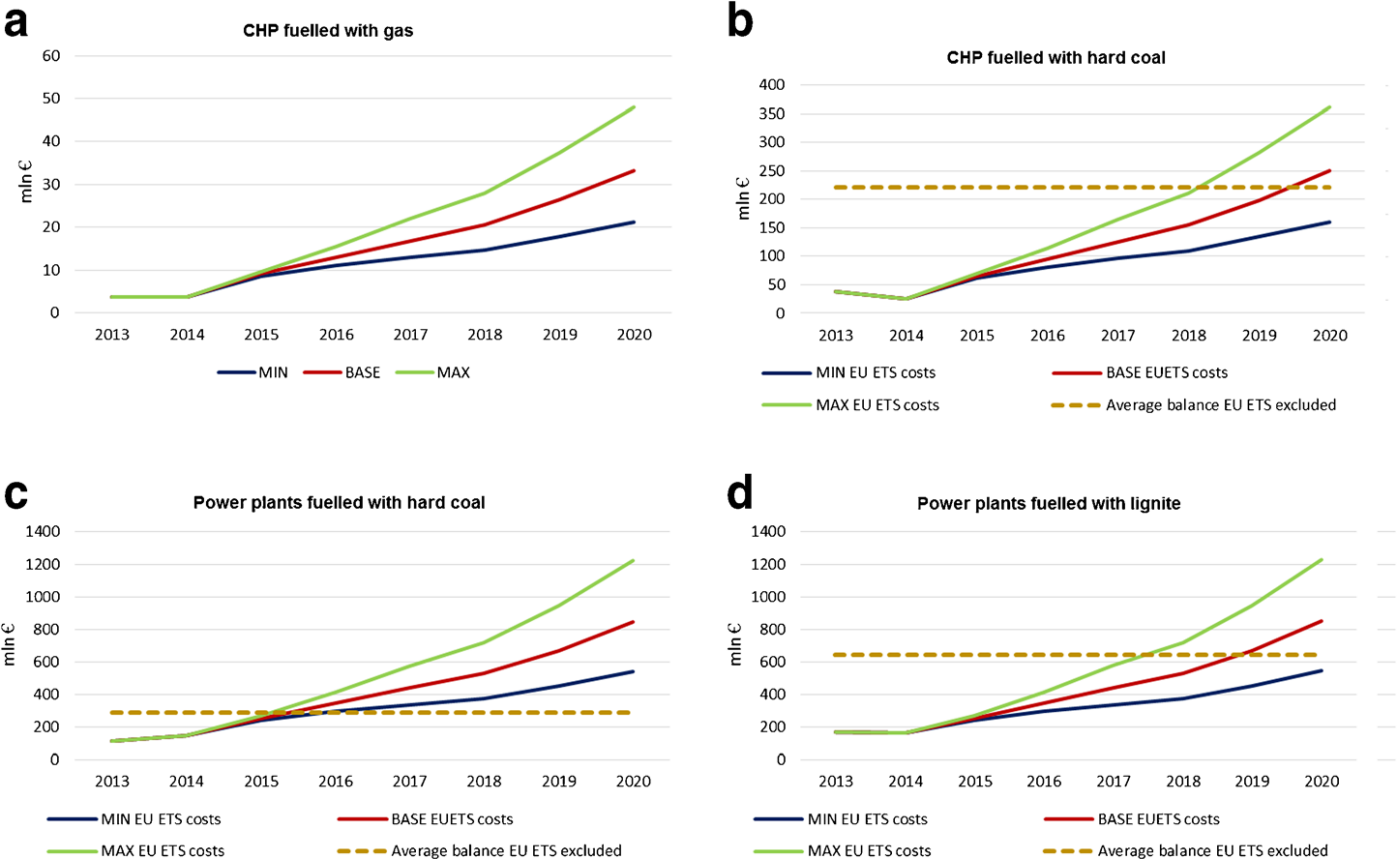
Plants profitability based on historical balance and forecasted EU ETS costs. Source: own study based on data from ARE S.A.

Projected costs of purchasing the missing EUA were counted by multiplying the yearly estimated shortage of EUA for a certain group by the EUA cost for the three scenarios (see Fig. 2). In order to determine a possible economic impact of the EU ETS participation, the projected costs were compared with the average historical, i.e. from the years 2008–2014, financial balances of the power plants. Since in this analysis the BAU scenario of the power plants was assumed, hence possible changes of other factors, e.g. fuel price, GDP fluctuations, energy demand, annual temperature change and technological progress, were not taken into account. It is possible that the EU ETS costs themselves reach the balance and thus make the conventional power plants not profitable (Fig. 14). The gas CHP plants are the least impacted by rising CO_2_ costs. For the other groups, the base scenario costs will exceed the average balance in 2019.

#### Discussion

When it comes to hard coal-fuelled plants, the difference between plants producing only electrical energy and CHP plants is noteworthy. The plants producing electric energy only proved a lot of inefficiencies. Their profitability levels were the lowest when compared with other groups and even reached the level below zero in 2013. When analysing the future, in regard to growing EU ETS costs, they showed the biggest vulnerability, and regardless of the EUA price scenarios, they may lose their profitability the soonest unless the energy costs increase. An assessment, similar to our short-term forecast, on profitability of coal-fired fleet in Texas (USA) concludes that *fundamental changes in the Texas electricity market are putting coal-fired power plants under increasing economic and financial stress* (IEEFA 2016b)*.*

The second category, i.e. CHP, managed to gain some additional revenue in the past years as it is not a big emitter and, assuming minor changes in their BAU scenario, is going to remain profitable in the next years. The results support the conclusion that gas-fuelled CHP plants, now the smallest installed capacity in conventional power sector, are the sources best immune to price changing regarding the EU ETS-related costs. Even the most severe scenario (MAX) of EUA price change will not affect its profitability to the extent that the CHP plants lose economic viability. They are also very operationally flexible, which is of great importance considering the growing share of intermittent RES and the lack of significant energy storage capacities in the power system, e.g. pump-storage plants. The only drawback is that they use imported fuel and, therefore, they make the country more dependent on import. However, it is certain that its share in the Polish energy mix should be increased especially given that the gas does not need to be entirely of Russian origin anymore, since the new LNG gas terminal in Świnoujście was put in operation in 2015. It takes LNG from the Middle East, mainly Qatar, but in June 2017 for the first time from the USA what shows a growing openness and flexibility in global energy markets.

Moreover, lignite-fuelled power plants should not be phased out, because even though they demonstrate the highest emissivity, they are very cost-effective. Their average profitability over the years 2008–2014 was the highest when compared with that in the other groups discussed. The analysis of the impact of rising EUA price on the profitability of these sources showed that assuming a base scenario of the price change, they would lose their economic sense in 2019; however, with the minimum (MIN) scenario, they would remain profitable. This conclusion coincides closely with the findings of Climate Analytics (2017) which demonstrates that market-forced shut-down time of lignite power plants in the EU is longer than for hard coal plants due to the higher profitability of the lignite ones. For example, the report predicts for Bełchatów (5 GW on lignite) the shut-down time for 2055 and market shut-down for 2027. Additionally, the lignite mines are owned by power generators what makes their economic situation better as compared to hard coal sector still mainly controlled by the state. However, the lignite sector faces a problem of fuel scarcity after 2030 when the existing lignite fields will be partly close to exhaustion that may lead to a drastic capacity reduction from current 9000 MW to around a third (Climate Analytics 2017).

Wholesale electricity prices in the EU reached maximum in the third quarter of 2008 and, apart from a minor increase in 2011, have been on the slope ever since. Prices have diminished by almost 70% since 2008 and by 55% since 2015 and in 2016 recorded the lowest level for 12 years. This diminishing trend is not however reflected in the retail prices which exhibit a rising tendency. It is explained by steadily diminishing “energy” component in the total prices which fell by 2.8% in the period of 2008–2015 whereas the” network” component rose annually by 3.2% in these years, and there was a significant increase of the share of taxes and levies from 12 to 32% of the total price. In Poland, “energy” costs were slightly higher than “network” costs (EC 2016e). The tendency is growing since RES component and costs related to stranded assets in the power sector are steadily increasing not being fully compensated by the diminishing costs of energy generation. The two opposite trends make the energy prices stable over the period of 2012–2017. The wholesale electricity prices in Poland count to the lowest in the EU. Comparing electricity prices for end users in Poland, it can be noticed that they are below the EU average and much lower as compared to the most expensive MSs. The price for industry in the second half of 2015 was €86/MWh whereas the EU average was €119/MWh and the peak in Italy €160/MWh and the UK €149/MWh. The price for households is controlled by the energy regulator and kept low to protect citizens, and it was €198/MWh, which is lower than the EU average €211/MWh and substantial lower than in Denmark €304/MWh and Germany €295/MWh. Therefore, the Polish society apparently benefits from the extensive use of coal in power generation. It cannot be forgotten that not all public subsidies and tax reduction in the energy sector are publically available which makes the economic assessment of actual energy costs highly uncertain. For example, data from the Organization for Economic Cooperation and Development show that Poland’s government supported its coal industry with more than $840 million in tax expenditures and budgetary support in 2011. Consumer and producer subsidies would make the number higher (Kowalski 2016). On the other hand, it is notable that according to the official EC database (EC 2016d), the state-aid expenditures (tax and fiscal measures) in Poland were one of the lowest in the EU amounting to €1 billion cumulative in 2008–2014, hardly comparable with Germany public support, in the form of tax allowances, tax advantages, direct grants and tax base or rate reductions, surging to €42 billion. This disproportion places Poland’s energy sector in much unfavourable, less competitive position against our neighbour. The EC warns that *in the North-Western Europe region (Central and Western Europe, Nordpool and the UK), natural gas prices and emission allowance prices (ETS) had stronger impact on the wholesale price level than in Europe as a whole* (EC 2016d; OECD/IEA 2014). The EU permanently experienced a high price ratio of gas to imported coal as it was 1.7 on average in 2008–2009, amounting to 2.7 in 2014–2015 and then falling to above 2 in the beginning of 2016. This decreases the share of gas in the European energy mix, although being accompanied by a diminishing share of coal gradually replaced by cheaper RES technologies (EC 2016d; OECD/IEA 2014).

Let us analyse some factors affecting energy prices in Poland. The first is the coal price. Due to the high proportion of coal-fired generation capacities in the Poland’s market, the impact of coal prices on electricity is high. For East European region, including Poland, the extent to which increasing costs of hard coal are directly passed through to electricity prices at the national power exchanges is the highest in the EU amounting to 70…80% (OECD/IEA 2014). Concerning the impact of electricity prices on hard coal, the IEA assesses *high impact, since mid-merit coal plants set the price in 80% of the hours due to renewable expansion and high-cost differences from gas plants* (OECD/IEA 2014)*.* Capros (2006) noted that *passing through opportunity costs depends on market power in electricity supply. It has nothing to do with the EU ETS system but depends on the ability of competition and regulation to limit potential rents.* The full complexity of the pass-through EU ETS costs on electricity prices is studied by Weishaar (2016) who explains differences between theoretical approach and empirical literature.

The second factor affecting energy cost is the cost of EU ETS participation. Pass-through of the costs of decarbonisation on electricity prices addresses the question to what extent the climate policy costs contribute to the rise of energy prices which may result in lower competitiveness and higher energy costs for the end users. EUA low prices of €4.5…6/t CO_2_ have not had a notable impact on the electricity market. It is estimated that the increase of the EUA price by one euro results in the wholesale electricity price increase of €0.2…0.8/MWh depending on EU markets (EC 2016d; OECD/IEA 2014). An extensive analysis on the EU ETS pass-through effects on energy prices is presented by Laing et al. (2014). They proved that pass-through cost in electricity can amount to 20…100%. Concerning the EU ETS impact on electricity prices, Lise et al. (2010) showed by simulation that in Poland, wholesale electricity prices are very vulnerable to increase of the EUA price. The pass-through of the EUA cost to electricity price in Poland is extremely high as the portion of allowance price of €20/t CO_2_ amounts to €19/t CO_2_. Such a high pass-through rate is caused by the coal-dominated power sector. The average for EU-20 examined countries, excluding Poland, was €10…13/MWh. This means 12…27% increase to the wholesale electricity price in the countries due to emission trading. Depending on the fuel prices, mainly the ratio of gas to coal prices, the additional EU ETS-induced price component can change the merit order, e.g. shifting marginal costs (€/MWh) of the Combined Cycle Gas Turbine (CCGT) technology before coal technologies.

Electrical energy prices in Poland have been rather stable over the years considered and do not exhibit a close correlation with EUA prices. Their impact on electricity wholesale prices is not traceable what indicates that power sector still finds a possibility for carbon cost compensation, e.g. in lignite-fuelled power stations being economically merged with lignite mines which give large margins for manoeuvres, or the cost is not yet pass-through to retail prices or is factored in, but at a low and barely visible price. The low electrical energy prices do not either create market signals for technological transformation. The impact is likely to remain modest unless the EUA price rises significantly.

## Long-term power sector decarbonisation

### Energy sector modelling

The short-term analysis of financial standing of power sector gives a clear warning that even with moderate EUA prices, there is a potential threat of losing profitability by some power plants by 2020 or soon afterwards. This conclusion is of operational nature and can be controlled by the government by ad hoc arbitrary decisions e.g. tax reductions and social obligation write-off, and further merges between coal and power enterprises. The current government’s investment plans both in coal and power sectors are likely to make the GHG abatement obligation not achievable in the long run. Therefore, the long-term strategic decisions on energy and technology mix in the power sector are unavoidable.

The EC takes its strategic energy decisions based on long-term outlooks produced by large-scale modelling at EU, regional and national levels mainly using PRIMES (Capros et al. 2014), supported by other packages, e.g. TIMES, TIMES-PanEu, GEM-E3, WorldScan, NEMESIS, Green-X and GAIN. The analysis of the impact of carbon prices on the electricity market carried by Capros et al. (2014) demonstrated that the EU emission reduction target set in the EU Roadmap 2050, i.e. the 80% GHG emission reduction target in 2050 as compared to 1990 emissions, is feasible with currently known technological options at low costs (lower than 1% of GDP in the period 2015–2050). Moreover, it is proved that delays in emission reduction until 2030 would have negative impact on the energy system. The full range of the scenarios used proves the need to replace coal by gas and RES. *All models show a continuous reduction of the share of coal in primary energy demand in the context of the decarbonisation scenario, despite the development of CCS after 2030.* The crucial role of CCS in the coal-based power sector is underlined by Eom et al. (2015) who claim that a low penetration of CCS would entail an extremely high deployment of other mitigation technologies, e.g. bioenergy without CCS, nuclear and solar, in the 2030–2050 period.

An analysis to check with what energy mix Poland is likely to meet the EU emission targets has been a challenge. Additionally, a preparation of new energy strategy, due in 2018, also requires a long-term energy demand modelling for Poland covering a number of scenarios with a different share of coal. Various scenarios have been for long discussed in extensive literature (Suwała and Labys 2002; Suwała 2008; Kamiński and Kudełko 2010; Kamiński 2009; Kamiński 2011). These scenarios, most of them accomplished a few years ago, assumed the never taken strategic decisions, e.g. commissioning the first nuclear plant in 2020, or they use outdated economic data, e.g. energy carriers or EUA prices, lower share of RES, a smaller role of energy efficiency in the national economy, and therefore do not fully comply with the current political and economic situation. The recent EC energy demand scenarios for Poland were developed in PRIMES in 2013 (EC 2013) and in 2016 (E3MLab and IIASA 2016). Neither nuclear technology nor CCS was envisaged. In EUCO30, one of the EC scenarios, with a target of 40% GHG emission reduction in 2030 compared to 2005, it is predicted for 2020 to produce 198 TWh of which 123 TWh on coal. The total capacity will amount to 43 GW of which 19 GW in coal. Thus, quite a large share of coal is still foreseen. The last long-term outlook for energy demand proposed by the Polish government was in 2014 as an accompanying analysis to the “Energy policy of Poland till 2050” (IGSMiE PAN 2013) dropped later in 2015 by the new government (Ministry of Energy 2015). A future domination of coal was taken as the main assumption. It was additionally planned to put into operation the first nuclear plant in 2025, the second in 2030 which now is not realistic. Wierzbowski et al. (2017) provided an extensive review of the current conditions of the power sector in Poland and discussed numerous simulations of Poland’s energy mix till 2050. They concluded that *Polish energy sector based on inefficient usage of coal must be transformed according to the requirements of EU energy and climate policy*. It is, however, noticed that such a transformation takes years and needs high investments.

**Fig. 15:**
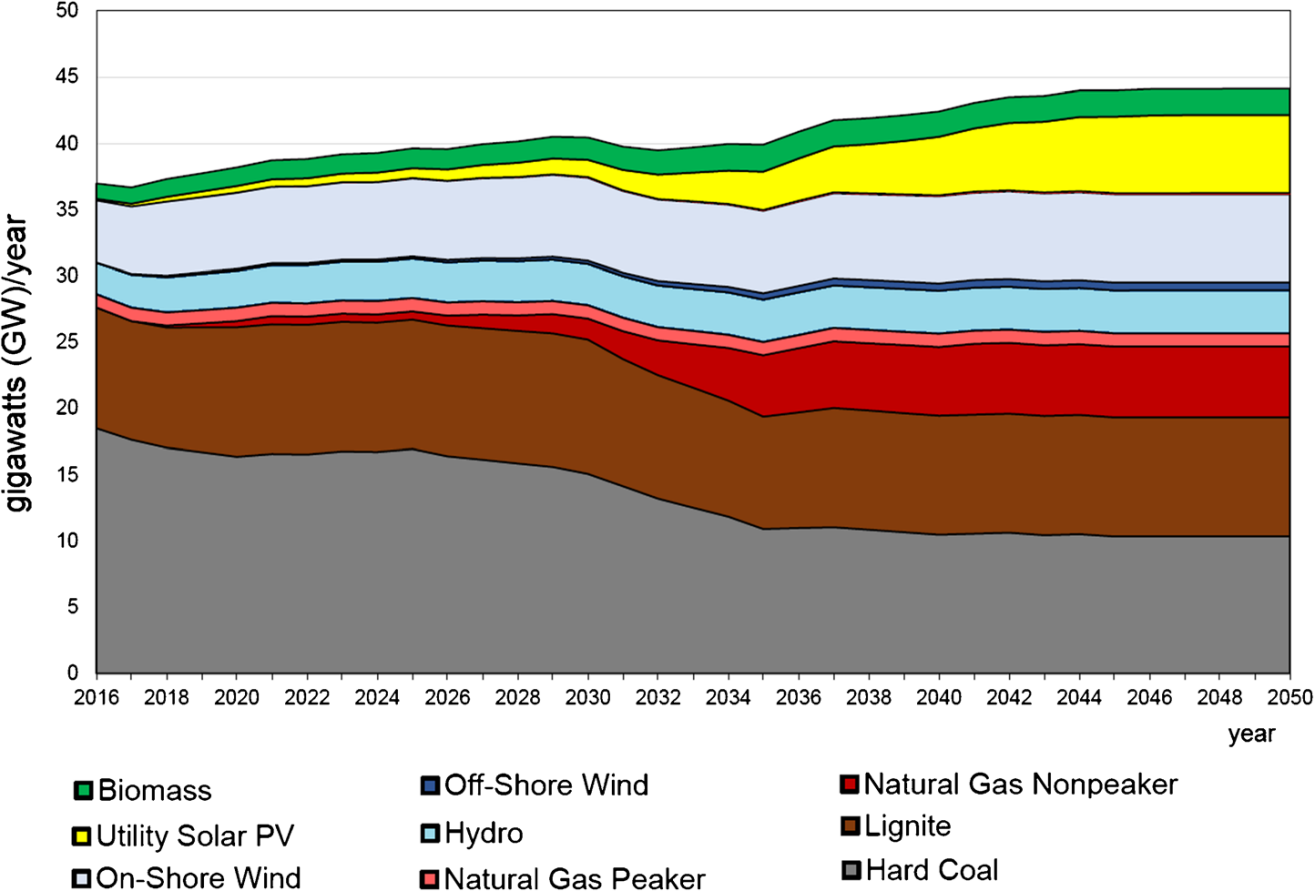
Power capacity by technology in the BAU scenario (without nuclear). Source: own calculation with the Energy Policy Simulator

**Fig. 16:**
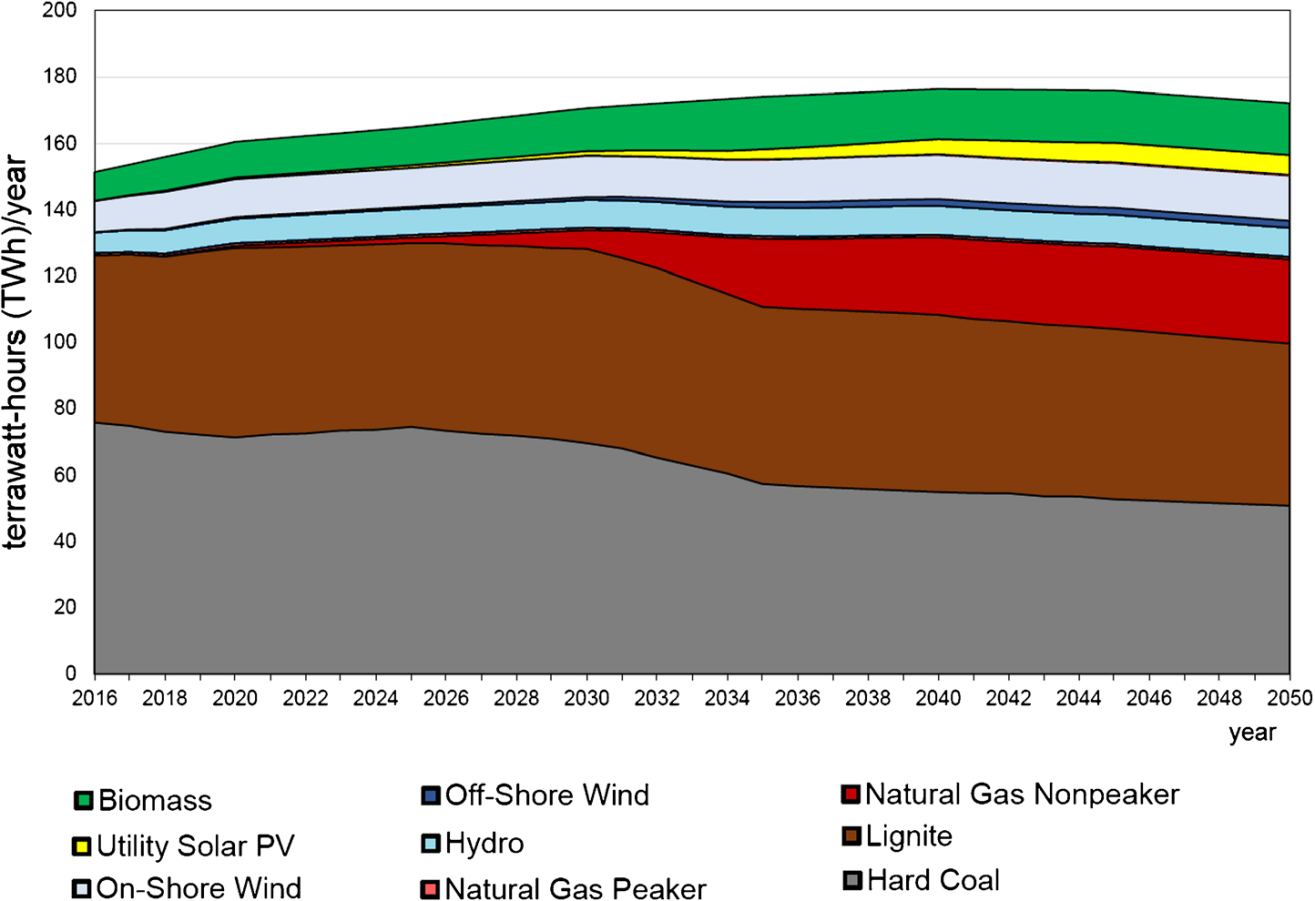
Electricity generation by technology in the BAU scenario (without nuclear). Source: own calculation with the Energy Policy Simulator

**Fig. 17:**
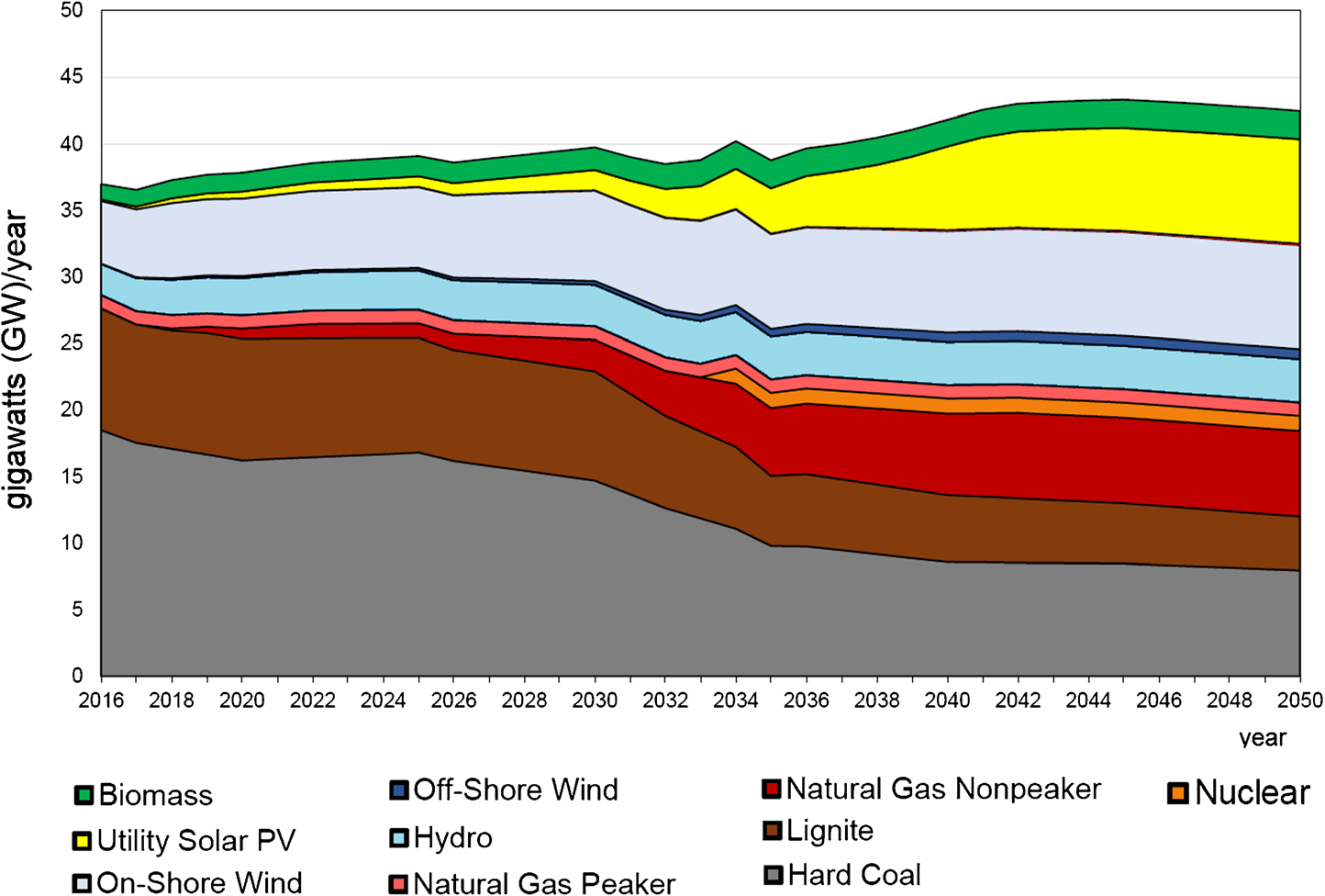
Power capacity by technology in the “40% Under 2005 Levels” scenario. Source: own calculation with the Energy Policy Simulator

**Fig. 18:**
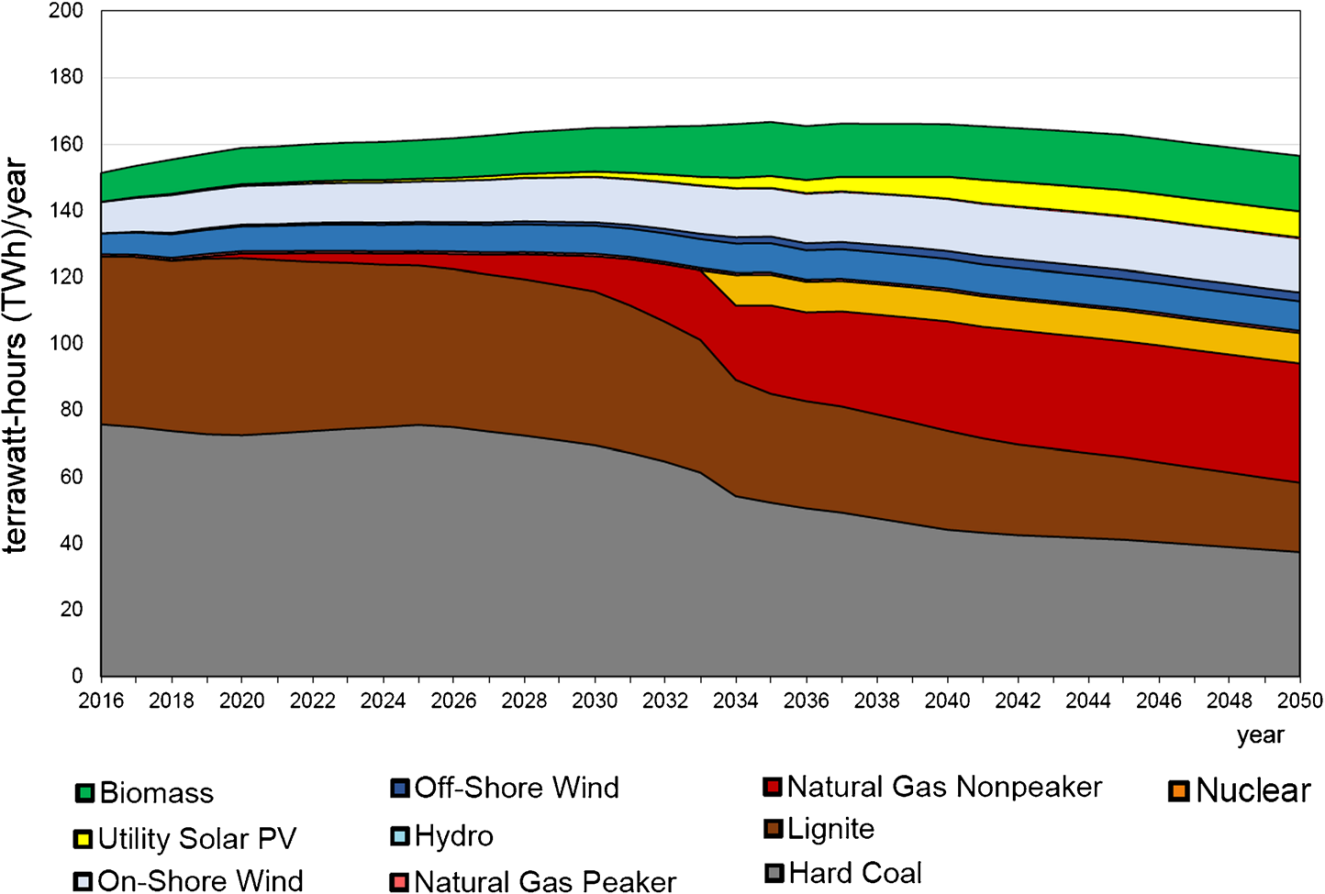
Electricity generation by technology in the “40% Under 2005 Levels” scenario. Source: own calculation with the Energy Policy Simulator

The very recent long-term forecast was made by the authors of this paper using the Energy Policy Simulator (EPS Project 2017). The Energy Policy Simulator is a computer model that assesses the effects of more than 60 energy and environmental policies on a variety of metrics, including the emissions of 12 pollutants and cash flow changes for government, industry and consumers; the composition of the electricity generation fleet; the use of fuels (coal, natural gas, etc.); and more. The model is designed to operate at a national scale and focuses on four major emitting sectors: transportation, electricity supply, buildings and industry. Some scenarios reaching 2050 were made to compare with the BAU scenario. The BAU scenario is built on the basis of existing governmental strategic documents or plans (Figs. 15 and 16). One of other considered scenarios aimed at meeting 40% GHG reduction in the whole economy in 2050 as compared to 2005 (Figs. 17 and 18) (thereafter “40% Under 2005 Levels” scenario), i.e. the same quantitative objective as in EUCO30 but to be met later, in 2050 (EPS Project 2017). The target was set as a reasonable goal for Poland in 2050 although it is worth noting that this 40% goal is hardly a half of the goal proposed by the EC for 2050. Similarly to the BAU scenario, this one takes into account as much as possible from the available governmental plans, and it is furthermore enhanced by adding some measures which are politically acceptable, technically accomplishable and economically viable. In the modelling, the EUA price path employed was very much close to the PRIMES trajectory (see Section 4.1).

### Discussion

**Fig. 19:**
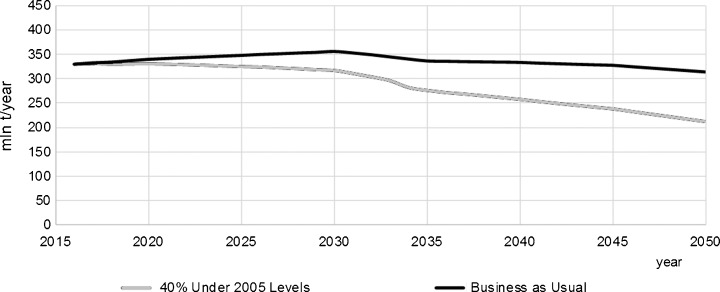
Total CO_2_ emission in Poland in the BAU and the “40% Under 2005 Levels” scenarios. Source: own calculation with the Energy Policy Simulator

The modelling shows that meeting this 40% reduction requirement means a substantial reduction of coal from the Polish energy mix in 2050—in hard coal from 18 GW in 2016 to 8 GW in 2050, and similarly in lignite from 9 to 4 GW (Figs. 15 and 17). The scenario sets the path to decarbonisation of the energy sector and concerning time constraints, e.g. duration of the investment process, and gives a strong impulse to start the restructuring as early as possible (Fig. 19). The reduction of GHG emission by 80% would entail almost complete extraction of coal. This conclusion coincides with the finding calling for closing all coal plants in Europe by 2030 if the EU wants to keep its Paris Agreement commitments (Nelson 2017a).

Power facilities in Poland are old—60% of turbo generators are over 30 years, 16% between 20 and 25 years and only 25% of units were built in the recent 20 years. As for boilers, it is that 63% is over 30 years and only 20% younger than 20 years. As a rule, power plants when ageing will exhibit substantial operational and maintenance costs, significant annual capital expenditures and a worsening operating performance. Paradoxically, the age of power capacity gives an alternative to invest in the most suitable technology under a criterion of long-term least costs to the society (IEEFA 2016b). Coal-based power capacity being near the end of its lifetime provides an economically viable option to commence a fuel switch and following technology replacement. Power plant operators undertake retrofitted works on old capacity to extend the life cycle of the coal-fired units which may bring temporary relief but cannot be economically viable and solve problems of GHG mitigation in the long term.

The minimum level of EUA prices to send sufficient market signal to switch from high emission trajectories to decarbonised economy has widely been researched. Bertram et al. (2015a) estimated that to limit a global mean temperature rise to 2 °C, the carbon price should vary between $16 and $73 per tonne of CO_2_ as in 2015. To enable a technological transition in the whole economy, they proposed to combine a price incentive of setting *a carbon price starting at $7 per tonne of* CO_2_*in 2015* with two other political instruments*—support for low-carbon energy technologies, and a moratorium on new coal-fired power plants to limit stranded assets.* Although the first requirement is at least partly covered by the new mechanisms in the EU ETS, e.g. the Modernisation Fund and the Innovation Fund*,* the latter is in a direct conflict with the current strong trend of investing in coal-fired power plants in Poland. Linares et al. (2006) demonstrated that in the long perspective, the EU ETS impact should result in fuel switching and expansion of the CCGT technology. Rogge and Hoffmann (2010) studied the role of the EU ETS in stimulating a diffusion of new technologies into the power sector. They conclude that such impact is visible in Germany in the large-scale, coal-based power generation technology, to which CCS technologies are added as a new technological trajectory. They also made interested findings that there might be EU ETS impact *on corporate* CO_2_*culture and routines may prepare the ground for the transition to a low-carbon sectoral innovation system for power generation technologies*. The report (World Bank 2017b) predicts that the explicit carbon-price signal required to meet the Paris-set climate objective shall be at least $40…80/t CO_2_ by 2020 and $50…100/t CO_2_ by 2030; assuming a supportive policy framework is in place. Nowadays, only the most optimistic and hardly realisable forecasts of EUA prices coincide with these figures. Valles et al. (2012) proved by a mid-term modelling of power sectors in nine MSs that prices of €15/t CO_2_ to €35/t CO_2_ are sufficiently strong to force switch from coal to gas technologies. Prices below €15/t CO_2_ are on the contrary to low as they insignificantly reduce emission although substantially increase electricity prices. It was also proved that even without free allocated EUA the thermal power plants remain profitable. Further sticking to coal plants will, accounting for their life cycle of 45…50 years, inevitably lead to the long-lasting carbon lock-in risks causing further political, economic and institutional entrenchment. On average, unlocking coal plants requires a carbon price of about $10…100/t CO_2_ which gives the average of about $30/t CO_2_, what is lower than for most technologies, e.g. e-mobility, but still more expensive than carbon emission prices valued by carbon taxes or CO_2_ reduction schemes in most countries. Gas power plants may be unlocked with a lower carbon price of $20/t CO_2_ (Erickson et al. 2015). Some recent analyses indicate that with more ambitious GHG reduction targets a further swift shift from gas to RES technologies may be required.

In this context, the case of the Czech Republic is interesting with the large coal-based power sector, now phasing out hard coal and considering reopening lignite mines. Modelling by TIMES revealed that the low EUA price (€7.5 in 2025) would lead to an increase in CO_2_ emissions, and the high EUA price (€80 in 2050) will make at least an 81% emission reduction in 2050 as compared to 2015. These emission reduction scenarios will give additional benefits of at least €38/t CO_2_, whereas scenarios resulting in CO_2_ increase are likely to bring additionally costs of at least €31/t CO_2_ on average over the entire period 2015–2050. It was concluded that in the Czech Republic, the development of the energy sector is mainly stimulated by the EUA prices which in turn determines GHG emission from the sector. That dilemma of reopening phased-out lignite mines is related to Poland where it is considered whether to build new lignite mines which could replace the old ones destined for shutting down after 2030 due to the exhaustion of existing fields.

These considered as well as other options of the long-term perspective exercised by the long-term modelling ensuring meeting GHG emission targets give many alternatives which may be converted to two main cases—to immediately launch nuclear projects or heavily invest into gas technology. Both options can be mixed with a certain dose of RES. The extensive use of RES is one of the alternatives to coal, in many countries, e.g. Germany, Spain or even China. IEA justly praises Poland *…the country has started a slow transition from coal toward more oil, gas and renewables* (IEA 2016a)*.* It is yet to be noted that the new RES act (2016), in the opinion of many, creates an unfriendly framework around renewable technologies and is likely to impede the development of RES, especially on-shore wind. Another option having been considered for decades is launching a nuclear project aimed at a construction of two nuclear power plants 3000 MW each. The decision has steadily been postponed.

Lecuyer et al. (2014) analyse the optimal timing of undertaking investment on the path to the transition from coal power to gas and renewable power under a carbon budget. They concluded that *optimal investment in renewable energy may start before coal power has been phased out and even before investment in gas has started, because doing so allows for smoothing investment over time and reduces adjustment costs. Gas plants may be used to reduce short-term investment in renewable power and associated costs, but must eventually be phased out to allow room for carbon-free power*. This finding seems to be a realistic solution for Poland; provided well-functioning EU ETS generates strong market signals to potential investors.

## Costs of transition of Poland’s energy sector

### Aggregated costs

The costs of transition in Poland’s energy sector are only roughly estimated, and the sources provide different assessments. The World Bank (2011) predicted by modelling that meeting GHG commitments for Poland is to be a costly process—costs to the economy will be the highest in 2020—1.4% of GDP as compared to 0.6% for the rest of the EU. Although then by 2030, the transformed low-carbon economy will support the development. Overall, this transformation will burden GDP by 1% each year onwards by 2030. The above average costs of economy decarbonisation are highly attributed to Poland’s dependence on coal in power generation. Several scenarios for the transition toward a low-carbon economy of Poland are discussed by Bohringer and Rutheford (2013). They also forecast that Poland will be more burdened than other MSs, since the transition costs are estimated at 1% of GDP compared to a BAU scenario, whereas the cost for the rest of the EU is about 0.5%. McKinsey’s report (McKinsey&Company 2009) identifies for Poland, by applying its world’s recognised standard methodology, a technical potential abatement of 236 Mt CO_2_e in 2011–2030, seizing that potential would equal to 31% emission reduction from 2005 levels or 47% from the levels expected in the BAU scenario. It predicts the cost of the whole economy transformation to tap the whole potential at €62 billion in 2011–2030—€92 billion for the required capital investment and €30 billion operational cost savings. The analysis also warns that a delayed or difficult implementation would lead to lower GHG abatement. According to the estimation by the Polish Electricity Association (PKEE) endorsed by the Union of the Electric Industry (Eurelectric), the overall capital expenditure needed for the Polish power sector to meet GHG emission reduction required as projected in a BAU scenario amounts to €65 billion by 2030. Besides, the Eurelectric assesses that the adaptation may cause in Poland a 20% rise of the electricity price (by €10/MWh) than otherwise projected by the EC proposal (Euelectric 2016). Another report estimates that 30% from Poland’s power sector will cost around €60 billion, and a 45% GHG reduction will surge the costs to €90 billion (EurActiv 2014). It is estimated that Poland can in practice reduce emission at the average cost of €10 to €15/tCO_2_, mainly by improving energy efficiency in no-power sectors (World Bank 2011). McKinsey estimated the abatement potential in the power sector at 120 Mt CO_2_ in 2030, at the average cost of €22/t CO_2_ whereas the average abatement cost for Poland was €10/t CO_2_. The system of energy efficiency obligation, the so-called white certificates, aimed at energy savings offers €32 for one MWh of energy reduction which is three times more than the cost of emission reduction in the case of the most emissive technology of lignite.

### Financing sources for energy sector transition

The role of public funding in the energy transition is crucial and cannot be overvalued. The unstable energy policy around meeting climate requirements creates unsecure investment policy for private investors shifting investment costs of new capacity to the public budget. The IEA claims that *states continue to play a key role in energy investments,* and *in 2016, 94% of global power generation investment was made by companies operating under fully regulated revenues or regulatory mechanisms to manage the revenue risk associated with variable wholesale market pricing* (IEA 2017). The share of public funds in generation investment amounting to one third in 2016 is reasonable and stable, while the network investment share of nearly 70% steadily rises. The IEA also warns that low-carbon generation is slowing. The EC (2016c) estimates that reaching its 2030 climate and energy targets will annually require about €379 billion for investments over the period of 2020–2030 allocated mostly in energy efficiency, RES and energy infrastructure.

Transition to low-carbon power sector requires large investment funds which exceed Poland’s national ability, and, in the public opinion in Poland, they shall be at least partly covered by the EU funds. Fortunately, the EU is determined to support climate policy by allocating a substantial share of its budget to climate-related projects, since 20% of the EU 2014–2020 budget supports climate-related projects.

Attractive, however demanding, financing sources for energy transition available over the financial period 2014–2020 are the European Structural and the Investment Funds (ESIF). Out of the total of €86 billion, around €19 billion will be invested in projects contributing to the Energy Union objectives in Poland. Another, not meaningless, option of financing energy infrastructure as a whole, i.e. enabling decarbonisation transition, is the usage of the European Fund for Strategic Investments (EFSI 2.0). The EC proposed to extend its duration till 2020 and increase its budget from €314 billion up to €500 billion. It had already been set that at least 40% of its budget shall support investments contributing to the commitments stemming from the EU’s Paris Agreement. ESIF has already trigged €154 billion on energy projects (23% of all projects financed). EFSI can be suitably combined with the Structural Funds, e.g. allocating €18 billion for energy efficiency for 2014–2020 (EC 2016a). In the biggest EU Research and Innovation programme “Horizon 2020”, expenditure on climate-energy research is to exceed 35% of the overall budget.

Supporting lower income MSs is explicitly written in the EU ETS reform objectives by providing funding to modernise energy systems (Skoczkowski and Wronka 2017). *Better support the transition in coal and carbon-intensive regions* is announced by the EC in the Winter Package (EC 2016c). There are different EU enabled options dedicated for this purpose. The greatest opportunity is created by the mechanism offered by Article 10c (see Section 3.2). To use the opportunity, in 2011, Poland submitted to the EC an energy sector investment plan embracing derogation (Article 10c) amounting to €45 billion. The EC in 2012 set the temporary number of free EUA for power sector at 405 million for the years 2013–2019 (which is the lion share of all 680 million EUA issued in the whole EU pursuant to Article 10c). The total market value of Poland’s share is estimated at €5…10 billion till 2019 depending on the current market value of EUA, e.g. in 2016 it was valued at €7.5 billion (EEA 2016b). As many as 77.8 million for 2013 and 72.3 million of EUA for 2014 were made available for the power sector (EC 2015a). The mechanism has failed to bring the expected results—the EC reports that in 2013, only about 10% of the investments triggered by Article 10c were spent to support low-carbon transition. Poland is among the countries misusing the funds subsidising the coal sector (Flisowska 2017). The allocation of the funds almost entirely in investments in existing coal base capacity came under high criticism (CAN 2014). To rectify the situation and cut off support for the coal sector, the European Parliament proposed the Emission Performance Standard that would eliminate all projects emitting more than 450 g CO_2_/kWh from the funding. The power sector will also have after expiration of the EUA auction revenues (Article 10c), the subsequent source of financing, namely the Modernization Fund (Article 10d). Between 2021 and 2030, 2% of the EUA, approximately 310 million (worth of €2…5 billion) will be laid by to establish the fund with contribution from all MSs. Additionally, Poland is to get €10…25 billion from EU ETS auctioning in 2021–2030 although there are opinions that this money should not be spent on modernising the power sector but rather on making Poland’s energy mix less GHG emissive and national economy energy less intensive (Bukowski et al. 2016). It is estimated that all the EUETS support mechanisms addressing the power sector envisaged by the EC for Poland, i.e. free carbon allocations and the Modernisation Fund, amount only to 10…15% of the predicted costs (EurActiv 2016).

### Discussion

The power sector in Poland faces the forthcoming need to operate in even a more hostile surrounding—coal prices are forecasted to stay low till 2050, making the global market more competitive and, therefore, putting political and social stress on conserving Poland’s coal-based power sector as the main coal purchaser; coal mines remain unprofitable despite public support and, therefore, unable to launch a technological transformation to increase productivity; EU climate policy will consequently exclude coal use, especially with unsatisfactory progress in the CCS; internal electricity market in the EU will strengthen market rules eliminating uneconomical plants; national market will be gradually opened for EU RES energy, e.g. wind and solar from neighbouring Germany, as stipulated by the Winter Package; distributed technologies will exert growing economic pressure on large-scale producers. Despite that, according to political declarations, the government plans to construct a new capacity of 12…15 GW (of which 4 GW are under construction) to replace the old units. The newly built coal stations, although using more energy efficient and lower emitting technologies, will soon face the same challenges as the old ones—they will lock the sector in deep coal dependence for decades.

According to the official statistics, the total public support for coal mining amounted to €16.4 billion in 2007–2015 of which €14.6 billion covered social obligations of the miners. In the same period, the coal sector paid similar amount of €16.1 billion to the national budget in the form of different taxes and fiscal obligations. In 2007–2015, the coal sector faced the problems of low productivity, overstaffing, ineffective system of remuneration resulting in high share of operational costs and high sensitivity to coal demand and coal price fluctuations. The three main coal producers embracing 27 coal mines recorded in 2005–2015 cumulative net loss of more than €0.27 billion. In the same period, the coal mine Bogdanka privatised in 2009 brought cumulative net profit of €0.35 billion. This comparison shows that a profitable operation of coal sector is possible even in the period of low demand and hardship in the global coal markets. In the opinion of the Supreme Audit Office (NIK 2017) at the end of the restructuring programme, the financial standing of the main coal producers was worse than in the beginning. The crisis was mainly caused by an unrealistic programme of coal sector recovering and unfinished process of privatisation in the coal sector.

Wynn tries to estimate the value of three brand-new coal-fired power plants put into operation in the Netherlands in 2015 (IEEFA 2016a). These power plants are dramatically losing their original valuation as well as projected investment return, and their owners may have to face additional losses. He claims that those cases build a barrier to any new-built coal power in Western Europe, while heralding headwinds for existing a coal-fired capacity. In the USA, one can additionally observe the economic and financial pressure from low natural gas prices falling precipitously since late 2008 and the following declines in the cost of gas-based generation. A recent report by Sanzillo (2016) points out that market forces have caused significant losses in the market value of coal-fired plants in Texas. It is reasonable to expect that a similar drop of financial value will occur in Poland, especially when post-Paris policy is successfully continued.

Additionally, it should not be forgotten that the energy sector in the EU, apart from GHG emission problems, also faces other environmental challenges mostly caused by the requirements of the Industrial Emissions Directive (Directive IED 2010) which comprises two old directives, namely the EU Large Combustion Plants Directive and Integrated Pollution Prevention and Control Directive. IED imposes significantly more severe standards of SO_2_, NO_x_ and dust emissions on combustion plants. Poland alone accounted for 45% of the heaviest EU polluters. The Transitional National Plan, which granted Poland a 4-year transitional period (2016–2020) to adapt to IED requirements, approved by the EC in 2014, will expire in July 2020 (Wood and Broom 2016). In April 2017, MSs agreed to more stringent limits on pollutants such as sulphur oxides (SO_x_) and NO_x_ from large combustion plants (EurActiv 2017). The power sector in order to meet the new limits by 2021 will either have to employ new technology to modernise existing coal-fired plants, limit operating hours below 1500 a year or even decommission the plant. The use of NO_x_ reduction technology would increase the electricity costs by €2…4 per MWh and SO_x_ abatement would add €6…7 per MWh. Thus, plants emitting both NO_x_ and SO_x_ above limits will have to increase generating costs by €8…11 per MWh. That additional burden is expected to increase the wholesale price of electricity in the EU in 2021 of €40/MWh by 5…30%. These costs when added to the EUA costs may make the electrical energy in Poland unaffordable both for industry and households (Wynn and Coghe 2017). The recent, entered into force in December 2016, National Emission Ceilings Directive (Directive NEC 2016) addressing air quality standards can in the long run further reduce the competitiveness of coal power plants as compared with other technologies (Climate Analytics 2017). It is assessed that this more pressing demand could force retrofitting or even the closure of one third of the Europe’s large-scale coal-based capacity. Reuters reports that Poland’s Enea, the third largest generator in Poland, is planning to spend up to €125 million to meet the newly imposed EU coal plant rules (Reuters Staff 2017).

Future steps whatever they are shall also take into account the provisions of the Winter Package, especially the proposed emission cap for a new capacity of less than 550 g CO_2_/kWh. EURACOAL (2017) points out that no coal plant can meet this standard unless fitted with CCS—the lowest emitting coal plants without CCS has the emission of 0.700 t CO_2_/MWh and many lignite plants produce more than 1 t CO_2_/MWh. It is assessed that 28 GW in Poland do not meet the requirement now (wnp.pl 2016). Also, the interpretation of the power market (Article 23 in EC 2016b) does not seem to allow for using this mechanism to restore the existing high emission coal-fired capacity in Poland. Additionally, the proposed Governance of the Energy Union (EC 2016f), imposing closer ties between climate and energy policies, is likely to exert its sustainable oriented impact on the whole power sector including EU ETS-related issues.

An emerging problem has been the growing reluctance of major international financing institutions to finance coal-based investments. It can be interpreted as a rapid response of the financing sector to the Paris Agreement. Buckley (2017) provides a number of examples where banking pulled out of financing coal-fired projects, e.g. Japanese, Chinese and South Korean financiers have resigned from financing three such projects in Bangladesh. Such institutions as the European Bank for Reconstruction and Development (EBRD), the European Investment Bank (EIB), the Deutsche Bank (BankTrack.org 2017; DB 2017) and many pension funds are changing their policies toward no investments in coal. Recently, in March 2017, a very spectacular decision was taken by the world’s largest state owned financial investor—the Norges Bank Investment Management who decided to withdraw its assets from some Polish energy companies involved in coal or coal-based power production (Norges Bank 2017). Those all financiers expect that high GHG emitters should have a transition strategy to a low-emission energy system. Although some other, despite official declarations, are still involved in coal sector financing, e.g. JP Morgan Chase, BNP Paribas financing a new coal block of 1000 MW in Poland (Ostrołęka). Such coal-biased policy, if consequent and spreading, may build a severe obstacle since it is questionable whether any Polish bank consortium can alone bare the financing burden and risk. This no-coal trend coincides with the Eurelectric declaration unexpectedly announced on 5 April 2017 that EU electricity utilities, except for Poland and Greece, will not build any new coal-fired power plants after 2020 (Nelson 2017b).

## Global dimension of Poland’s case

The role of coal in the modern world is contradictory—it enables the development by providing cheap energy, and it threatens that development by having negative impact on climate. The future of coal in the power sector related to the GHG abatement is also a subject of worldwide discussions (Akimoto et al. 2014). Coal, according to the IEA, will remain the main energy carrier for decades with a predicted fall of coal share in the global power generation mix from 41% in 2014 to 36% by 2021 (IEA 2016b). The diminishing investment trend in coal-fired plants is visible now as in 2016 nearly 20 GW fewer were commissioned following climate concerns and the emergence of overcapacity in some markets. The final investment decisions for new coal-fired power plants taken in 2016, totalling a mere 40 GW globally, indicate low interest in future coal technology investment once the current construction trend ends.

The EEA (2016c) predicts that solid fuels share in electricity production will shrink from 97% in 2014 to 80% in 2030 in the Central and East European (CEE) countries, whereas gas will increase substantially to become responsible for 20% of the production of electricity by 2030. The EEA concludes that there is no risk of carbon lock-in for the CEE countries which seems to be corrected only for the whole block of countries but not for Poland where continuing the investment in coal capacity makes the risk real. A danger exists that erroneous investment decisions on power capacity, concerning the future fuels and technologies used, may lead even in the short term to over- or under capacity at a national power grid level. A possible shortage of energy supply may easily be covered by energy import, provided sufficient interconnection infrastructure exists, e.g. the EC wishes to meet the currently adopted 10% interconnection target by 2020 with options to find cost-effective ways to increase it to 15%. A completion of regional energy markets with high interconnector capacity poses a threat of losing competitiveness of national power sector due to lower energy prices in the neighbouring countries. In Poland, such a market transition is a subject of political discussions in the light of cheap energy supply from neighbouring countries in which RES-produced energy is heavily supported. Future energy mix and technological issues in the long-term perspective are essential and can be decided upon only when decisive political decisions, for example on coal phasing-out, nuclear option, RES development rate, energy efficiency aggressive policies, are in place. The lack of firm political decisions on social and economic development makes numerous long-term energy outlooks rather academic topics than strategically useful plans. These forecasts shall also address the social issues in coal-intensive regions to enable taking the right steps to mitigate them.

Poland is not alone in its struggle to reform the power sector based on coal. Goodman et al. (2016) summarised research on the role of coal, mainly investigating the cases from India as industrialising country, Germany being a post-industrialised country in energy transition and Australia with its resource-dependent economy. Poland’s lesson can be regarded as complimentary to these considerations as a case of a country heavily relying on coal and a subject to the highly demanded climate policy of the EU. Similar problems occur in the USA, China, Australia or India and in many other countries worldwide. Reforms in these countries are being strongly driven by climate change policies (World Bank 2016). There are also a number of other factors that are more decisive in taking investment decisions in power sector than low EAU prices (Hoffmann 2007). Nepal et al. argue, basing on data from 28 countries including Poland and covering years from 1990 to 2012, that the Kyoto Protocol was not the main drive in mitigating GHG emission per capita. They point rather at increased economic efficiencies resulting from mixture of market reforms that contributed to emission reduction. This conclusion supports, apart from direct emission curbing instruments, the need of reforming all sectors with a substantial relevance to the climate change. The ineffectiveness of the EU ETS in phasing-out coal-fired power station is also, despite all the reforms undertaken, one of the main concerns in the after-Paris policy (Climate Analytics 2017).

China reaffirming its climate policy seems to be on the path of curbing the role of coal in the power sector and aims at reining in its overcapacity. The country announced the suspension of 104 planned coal-based power plants with a total capacity of 120 GW, of which around 54 GW are from projects under construction. Although China remains, with 21% of the global total investments, the leader in energy investment, yet in 2016 a 25% decline in commissioning of new coal-fired power plants took place (IEA 2017). The USA generated 30% of its electricity with coal in 2016, much down as compared to over 50% a decade ago. Also in India (IEEFA 2017b) and Bangladesh (IEEFA 2017a), the shift away from coal has been much swifter than predicted although the latest report (IEE 2017) predicts that coal will remain a dominating fuel in India’s baseload power till 2047. In India, a stricter regulatory framework, especially addressing the environment, is also of concern. To meet the Paris commitments, India will have to increase the supercritical technology from the current rate of 11.5 to 50% by the time. Even some countries of the Organisation for Economic Co-operation and Development (OECD) plan or have under construction of a new coal-fired power capacity, e.g. Turkey (74 GW), Japan (22 GW), South Korea (20 GW) and Poland (9 GW) (Endcoal.org 2016).

African countries lack, in opposite to the developed countries, a strong regulation in the energy sector. A programme New Deal on Energy for Africa (ADBG 2017) aiming at creation of a supporting policy environment to foster adequate private sector interest and sustainability has recently been launched. Reluctance of major international financial institutions to coal investments is likely to increase, which may put a new barrier to invest in coal-based capacity, especially in developing countries. Developing countries should follow the leaders in transformation, despite their possibly different starting position.

In the EU, climate policy-related aspects seem to be a steady and long-lasting component of the future economic development—*overarching priority for the Commission over the coming years* (EC 2015c). Such EU approach, very much oriented on economy decarbonisation, provides enormous challenges for those MSs which still use coal. Some of them like the UK, Germany, Belgium, Portugal or recently Finland (Morgan 2017) have adopted their ways to solve the problem of coal, and they are on the path to coal phase-out; some, like Poland, the Czech Republic, Romania (World Bank 2015) and Slovakia, are still coping with this socio-economic problem of national importance (Climate Analytics 2017). Bertram et al. (Bertram 2015b) point out that near-term climate policies allowing for larger emissions may take up more of the EU 2010–2030 long-term GHG emission abatement budget which will result in higher budgetary expenses and the urgency of reducing GHG emissions after 2030. Moreover, a weak near-term GHG emission target set by less stringent policies encourages investments in additional coal-fired power stations. Unclear future of the energy sector in the EU, caused by constantly rising climate-related demands, is one of the reasons why EU investments in energy are weak—with an investment decrease by 10% in 2017 as compared to 2016—mainly as a result of lower renewable investment. The EU countries, although fairly successful in the GHG abatement from the power sector, are not free of challenges. Many argue that the adoption of the proposed Emission Performance Standard—EPS 550 (550 g CO_2_/kWh) may enforce a start of gas phase-out in the EU. Even gas peak-hour capacity may be eliminated. The Eurelectric (2017) claims that the fulfilment of the EPS 550 would result in a 40% rise in gas consumption for the EU power sector in the period of 2020–2040. Meeting the EPS 550 objective would cause extra EU power sector investment costs of around €50 billion between 2020 and 2040, mostly spent on additional GHG emission reduction. Gas consumption in Western Europe would increase by 100 TWh per year, resulting in annual gas increase above 400 TWh until 2035. For Eastern Europe alone, it means an increase of gas demand by 90 TWh per year in 2025 to 160 TWh per year in 2040. As much as 50% of the increase of annual gas consumption in 2040 would take place in Poland which would dramatically turn down national energy security.

Some states in the USA are also heavily coal dependent—in Wyoming, nearly 90% of electricity comes from coal; in North Dakota, 80%; and in Colorado, 60% (2015). Kowalski (2016) makes an interesting comparison of the State of Ohio and Poland by noting that similar to Poland, a bulk of Ohio’s electricity comes from coal. Ohio was the tenth largest coal-producing state and the fourth largest coal-consuming state—in 2015, coal-fired power plants produced 59% of Ohio’s energy. Although coal still accounts for a majority of the state’s electricity generation mix, the share is down from almost 86% in 2006. In the same period, Ohio’s natural gas share in electricity production increased from less than 2 to 23%. She points out that the plunge of the US coal production in 2015 to 900 million tonnes set a 30-year low and was a 10% fall from 2014. The drop was accompanied by seeking bankruptcy protection by large coal companies, e.g. Arch Coal, Peabody Coal and Patriot Coal. This could be a warning to all coal companies operating in the competitive market of low-price energy carriers mostly caused by cheap gas. In Europe, including Poland, this is not the case—gas prices are substantially higher than in the USA and energy prices are increased by the EUA costs. Some blame too strict federal law on GHG emission for crippling the competitiveness in the US coal industry, and this is exactly the same argument circulated in Poland. The other reason is the fact that coal-fired power plants in the USA are older on average than modern competing technologies of electricity generation, and their efficiencies are lower than those of newer plants (SourceWatch 2017). The decision of President Trump to leave the Paris Agreement and review the Clean Power Plan (EPA 2017), having in back the restoration of the coal industry, makes the coal market and power technology development paths difficult to predict and the market more uncertain. This decision, although widely contested by many, e.g. Stavis (2017), opens a room for a new round of the worldwide discussion on the coal perspective. Low prices of coal in the world market affect the coal sector very much similarly in the USA and Poland. The economic difficulties in the global coal market forced Poland’s government to decide in late 2016 to merge some unprofitable hard coal mines with relatively well-doing power plants. Consequently, the economic standing of the latter is likely to get worse.

## Recommendations

The global combat with the climate change inevitably entails a need to rethink the future role of coal in power generation. It applies to countries with a large coal sector but as well to large coal importers which are now at the crossroads choosing future energy mix and power technologies. The global transition to clean energy is also in the interest of poor populations striving to lift themselves out of poverty which requires confronting the climate change in many aspects, e.g. natural disaster threats, water scarcity, land use, deforestation. For all of them Poland’s lesson on transition of coal based energy sector may be useful - it may serve as an example of a country historically based on coal and finding itself under pressure of the world’s leadership of the EU in climate policy. The article, by giving a thorough analysis of Poland’s case, formulates a number of recommendations applicable for coal relying countries inevitably facing the energy transformation.

### Completeness and timing of economic reforms are essential

Reforms in energy sector, embracing coal and power sectors, shall be an essential element of the country’s transition toward a sustainable model, aiming at low-carbon emission and based on circular economy principles. Economic reforms must embrace the energy sector from the very beginning to decide upon the optimal energy mix and scale the energy capacity to the future demand avoiding stranded costs. The alignment of economic, energy and climate policies should catalyse the transformation of the country. The lack of correlation between the reforms in the energy sector with reforms developed in other sectors inevitably leads to higher costs for society. A similar negative impact will bring partial, unfinished or delayed reforms.

The transition must be rather a well-planned evolution than a series of ad hoc taken political decisions, mainly forced by the rapidly changing global climate policy or short-time unstable situation in energy markets. Phasing-out coal shall be planned in a way securing a smooth transition to alternative fuels, especially in coal-extraction areas where the job loss and a breakdown of coal tax revenues in local economy are likely to happen. Reforms should envisage a compensation for the most vulnerable social groups or even business sectors. Opening of new businesses to offset the job loss in the coal sector and social support programmes is necessary to lower social stress. A change of value in the whole energy chain must be recognised as it determines the willingness of private investors.

Poland’s case shows that the lack of political will to undertake a truly deep energy reform when the whole economy transformation began may lead to postponing the reforms for decades. It results in high public costs difficult to be overturned even in the long run, e.g. subsidies to the coal sector, unsolved social problems in coal extraction regions and high environmental pollution.

The switch from coal to other technologies shall be complemented by activities that abort and limit economic and social emission abatement costs. Only a suitable combination of such measures may bring desired results in a manner accepted by the society. Such reforms are unavoidable and have successfully been carried out in many highly developed countries of the OECD, e.g. the UK or Germany, and have recently been announced in France or Finland.

### Political decision must be brave, non-sectorial and non-technology-oriented and remain stable

Political weakness concerning energy sector following a threat of social unrest in coal regions, ill-defined conditions of national energy security based on coal self-sufficiency or even private interests of politicians is a classic example of why reforms tend to be neglected or delayed in the energy sector. Reaching a political consensus on clean energy transition may be a challenge but is a prerequisite for launching reforms and such an agreement shall not be subject to any political contest and trade-off. The costs of the low-carbon transition and its social implications may be high, especially in the short perspective, but then they will become profitable to the society. Energy transition must receive the support from citizens which in turn will encourage politicians to undertake difficult reforms. Predicted negative impact of climate change has played an important role in mobilising public support for energy transition in many countries. Although, it is still not easy for politicians to communicate to the society, especially to the most afflicted groups, the need of covering costs of such transition.

### Long-term economic and social benefits shall decide

There are a number of international activities supporting clean energy transition globally including those aimed at helping developing countries to meet their Paris Agreement commitments. These activities usually provide financing instruments which can be used for facilitating the transition. If used systematically and effectively, they can alleviate financial barriers of the transition especially at their initial most opposed stage enabling to gain momentum and prove its benefits to the society. The first step in estimation the real costs of heavily coal-dependent power sector is to establish independent cost monitoring of coal sector embracing all public support and costs due to GHG abatement. It is a rule that substantial public support distorts the energy market and is not transparent to the public. A short-term economic balance of power plants accounting for costs of GHG emission reduction may give an early signal for the urgent need of energy market reforms. Delays in taking political decisions will unavoidably lead to higher social costs, perhaps only shifted in time. Long-term modelling provides some guidance on future energy mix to meet the GHG reduction goals in the most economical way. The second step is to assess the cost of coal dependence in the long run till 2050. A low-carbon scenario shall be compared with other options covering a different mix of gas and RES, and where politically acceptable, also with a nuclear one. Real GHG abatement costs, these in place or envisaged, and the needed public support to counteract the emission shall be fairly modelled in the long-term energy scenarios.

### The right incentives for energy transition shall be clear and acceptable for all stakeholders

Governments shall set a favourable and coherent system of economic incentives to launch the clean energy transition and attract private investors to complement public funding. The price incentives to power sector decarbonisation should be sufficiently strong and stable in time to launch investments. Moreover, they should not give dispersed signals which may mislead investors or give them false hopes that the transition is not necessary. Power sector reforms and the GHG emission abatement mechanism shall give clear long-term signals for investors to limit business risks. The costs and risks of transition, for instance closing coal-fired plants, should be balanced with gains and allocated to all stakeholders, not only to end users. It should be noted that coal-based power plants are compelled either to shut down or to make significant GHG abatement retrofits. Decarbonisation strategy shall enable to raise both public and private funding for the energy transition**.** Harmful market distortions must be removed, and market mechanisms employed to set the level playing field. Carbon pricing, e.g. emission trading systems, carbon taxes and tax exemptions, shall give clear investment signals to launch the transition and secure a return on investment. There are a number of methods which the government may use to secure a smooth energy transition e.g. power market development, combination of energy efficiency and RES policies, demand side management, legislative and regulatory measures, carbon taxes and EU ETS-like abatement schemes. Additionally, fossil fuel subsidies shall be removed according to the commitments made under the Paris Agreement and within G20—the international forum for the governments and central bank governors—*reaffirming their commitment to ensure access to affordable, reliable, sustainable and modern energy for all* (EC 2016g)*.* The G20 declaration is in sheer contrast to the estimation that G20 country government subsidies for the production of fossil fuels amount to €416 billion annually (HEAL 2017). The group with the seven largest advanced economies in the world (G7) has also failed to endorse a common statement that referred to the Paris Agreement (EUobserver 2017). On the other hand, also subsidies to RES and energy efficiency investments should be removed in the long term when their competitive markets have been established.

### Wide international context shall be not forgotten

Transition to a low-carbon economy has also its international dimension and as such it should be an integral element of political and economic relationships in bilateral and multilateral contacts of regional or even continental dimensions. Decarbonisation trends shall be internationally co-ordinated to cover at least regions, e.g. in the EU to embrace the whole community. Such a large geographical coverage can provide least-cost trajectories to low-emission power sector. If high-emission islands remain, the decarbonisation plans in other countries are likely to be impoverished which may demotivate the front runners. The IEA in its Paris Agreement-compliant scenario claims that unabated coal power plants must be closed in the EU in the beginning of 2030s. The developing countries shall secure their growing energy demand in a way complying with the global climate policy. Meeting the energy transition goals in a cost-effective way, partly by allocating available financing e.g. envisaged by the Paris Agreement or EU ETS, is a priority both for donors and recipient countries. The rationalisation of the EU funding landscape and an active synergy between climate abatement efforts with EU structural funds is also recommended to enable MSs and regions to cope with challenges posed by the climate change. The time and way of phasing out coal dependency differ from country to country depending inter alia on national fuel resources, RES potential and public support for a nuclear option to secure the changes needed in the energy mix.

### Energy mix and technological transition shall be carefully designed and matched and should remain stable for long

Technological transition in the power sector must take place in Poland as well as in the majority of countries to achieve their GHG emission reduction objectives. The power sector transition is strongly environmentally and technologically driven with political ambitions still lagging. As a consequence, the power industry is undergoing a tremendous change across the whole value chain which in the market perspective leads to losing economic value of coal fleet due to environmental costs and a gloomy perspective in the post-Paris environment. Disregarding technological progress and persistent investing in obsolete technologies not meeting environmental objectives would be a waste of time and resources. The progress in some technologies, e.g. in RES and energy storage, has turned out to be much faster than expected, whereas the CCS progress has disappointed. A decision on the optimal set of generating technologies shall also take into account the main changes ongoing on the demand side to avoid stranded costs, e.g. increasing energy saving potential due to technological progress, change of patterns of energy use in households and services, alternate pricing schemes, energy management progress in industry and low-energy intensive technologies diffusion in many developing countries in the process of their industrialisation. To lower the risk in power business and to have more freedom in taking investment decisions, some power sector enterprises split assets between the “old” legacy assets and the “new” types of business, e.g. RES, energy management on the clients’ side or energy services. Technologically enabled coal phase out should be accompanied by supporting regulatory and market reforms facilitating a smooth energy transition.

### Social issues are of predominant importance

Despite the common public opinion, the closure of coal-based power plants is not entirely caused by the coal-hostile regulation, but also by the economics tilting in favour of gas-fired plants and RES. Here, citizens with their mobilisation and involvement to take more responsibility for energy sector development are of key importance. Unsolved social issues related to coal phase-out seen from the time perspective to be the main obstacle in processing deep reforms in Poland’s coal sector. In the whole discussion on the future of coal, e.g. nowadays in the USA or Poland, it should fairly be mentioned that there are voices calling for reinvesting the coal sector (Sanzillo 2016). It may be a signal that societies feel in debt to coal miners and their families. Therefore, seeking solutions in countries with a still active coal sector only proves that social problems shall not be disregarded. Healy and Barry (2017) claim that *political action by civil society will be required to accelerate the phased ending of the fossil fuel era. More than that, it must end it in such a manner that the transition to a low- or post-carbon energy future minimises injustices of that transition and maximises its democratic character.* Attractive schemes of pensions for miners at the nearly retirement age, creation of jobs in new non-coal sector industries and services accompanied by trainings in new skills are the routine elements of social reforms. It is, however, often neglected to carry out the process of social adaptations in the way that gives the out-going miners a feeling of serving the society well. Closing mines in the atmosphere of defeat and of low social usefulness of miners will unavoidably spur social protests and stress. Proceeding of the reform starting with a “white book” to estimate the historical benefits and costs of the coal sector provided to the society would be a fair approach. Co-operation with local public authorities in coal regions is essential to gain support and reliability. The EC (EC 2016c) suggests to *provide guidance, in particular for the access to and use of available funds and programmes, and encourage exchange of good practices, including discussions on industrial roadmaps and re-skilling needs, through targeted platforms*. The involvement of all social partners in coal sector transformation is a key to success, and the role of trade unions is crucial. Establishing a dialogue between trade unions, government and private owners to seek consensus acceptable for all parties at moderate costs is an essential element of the reform. The lack of talks, entrenching of parties and unwillingness to understand common long-term interests may block the progress for years and conserve the unhealthy economic and social relationships in the coal industry with the negative impact on the whole country. Human resources with their commitment to the transformation objectives, possessing knowledge and practical skills in implementing energy transition rules into practice are often undervalued and hence become a missing element of a successful transition. A close observation of economic balance of individual energy enterprises, e.g. coal mines or power stations, may give arguments for the necessity of reforms. The indications of losing profitability, as demonstrated in this paper, were the main signals sent to politicians in Poland.

## Conclusions

The aim of this work was to determine the impact of the EU ETS on Poland’s conventional energy sector which is exceptionally dependent on coal. It provides analyses in two different approaches and time scales—in detailed economic terms in the years 2008–2020 and in the macroscopic scale in the long run up to 2050. The paper provides a short-term estimation of climate policy costs to the existing coal-dominated power sector. The estimations were based on 2008–2014 data which served as an input for the forecast analysis (2015–2020). It was proved that the financial standing of some coal-fired power stations may substantially worsen, provided the EUA prices increase as it is being planned by the EC. Furthermore, the conclusions indicate that 2020 shall be regarded as the deadline to commence a new phase of energy transition coinciding with the new EU ETS trading period. Before that date, the new energy strategy shall solve dilemmas on the future energy mix and the following structure of the power sector at least till 2050. It was also shown that in the long perspective, till 2050, to meet not very much demanding GHG emission reduction (40% in 2050 against 2005) will enforce retiring of a large share of coal-based capacity in Poland. The goal of 80% GHG reduction in 2050 demands almost an entire drop of coal in power generation. A recent report on coal-fired power sector in the EU, presented from the perspective of fulfilling the EU Paris commitments, forecasts even an earlier shutting down of Polish coal power stations due to their low market competitiveness (Climate Analytics 2017). Summing up the current Polish situation, it can be concluded that the EU ETS does not appear to have had much effect yet, either on electrical energy prices or on generation technologies. Technology remains invariant to EU ETS partially due to low EUA prices being well below the technology threshold and, therefore, not able to force technological breakthroughs, e.g. switch from coal to gas/RES. Additionally, the politically induced support for coal sector strengthens the dominance of coal.

Comparing the situation of Poland, still having a large share of coal based power sector, with other EU countries, it can be noticed that while others, e.g. Germany, Italy, Portugal or Spain, have clear plans to phase out coal, Poland, as the only EU country (except for Greece), stubbornly sticks to the development of the coal-based power sector. A political inability to adopt the energy strategy poses a threat that high energy prices or even power shortage may pose a barrier to Poland’s economic growth, the first symptoms of which energy users experienced in August 2015 when deep cuts of power supply to large industrial users were ordered by the Transmission System Operator. The risk of capacity shortage is growing due to the country’s delay in replacing dirty coal capacity, reluctance to support RES and postponing political decisions on launching a nuclear programme.

The lack of strategic planning and coherence in the Polish energy and climate policies are conspicuous. In practice, any deep restructuring process in the energy sector encounters a barrier of political dogma—an unquestionable and dominating role of coal in planning energy future whereas environmental objectives are perceived as second-order priorities. The gap between the defined strategy for coal-oriented investments in power sector and the climate objectives is not likely to get smaller. Thus, mostly due to political decisions, the energy sector is rather in the state of being coal captive. Despite the market signals, the government keeps on funding the mining sector regardless of the fact that it lost its profitability years ago. Moreover, some new power plants running on coal are being under construction despite the requirements of the EU climate policy. The recent idea to merge power companies with some unprofitable coal mines seems a time gaining solution that will just pass the problem from the government itself to power companies. The plan, announced late 2017, to construct two new coal mines is also striking. There are voices that Poland’s current political leadership now promotes coal as a means for continuing independence from external influences (Kowalski 2016). The IEA (IEA 2016a) has recently called upon the Polish government to *establish a clear vision for the coal sector, consistent with the new long-term energy strategy, based on a fair assessment of costs, transparent accountability and elimination of cross subsidies between power producers and coal mines, and provide the private sector with opportunities to develop and compete in the market.*

The threat of losing profitability by part of the power sector shall give a warning to politicians as to what technologies should be developed and whether coal is an economically viable option for meeting national climate and energy goals. On the other hand, the results obtained can be helpful for the energy sector by providing arguments proving the risk of losing profitability and calling for immediate actions from the government. For ambitious mitigation strategies, the period 2030–2050 is of critical importance, since it is then when the most rapid shift to low-carbon technology will take place (Eom et al... 2015). Therefore, it is so essential for decision-makers and investors to avoid the carbon lock-in in the power sector. The short-sight temptation to invest in coal-fired power plants, compensated by relatively inexpensive operational costs, would over time lead to political, economic, environmental and social factors that will make it hard to move away from, or “unlock” carbon (Erickson et al. 2015). Poland requires to resume a political dialogue on the future of coal—it is essential to send a strong message to citizens that the coal industry remains in a deep and permanent crisis, not because of the EU climate policy, but because technological progress has been making it possible to get energy from unconventional sources, e.g. the sun or wind, in a cheaper and cleaner way, and the shifts in global energy carrier markets. The period of “ineffective” functioning of the EU ETS may be a blessing period for Poland’s power system offering time for taking strategic decisions and restructuring. The time is going to end after 2020 when the new rules of EU ETS functioning come into force and the Winter Package reforms are accepted.

## References

[CR1] ADBG – African Development Bank Group (2017) Light up and power Africa—a new deal on energy for Africa, African Development Bank Group https://wwwafdborg/en/the-high-5/light-up-and-power-africa-%E2%80%93-a-new-deal-on-energy-for-africa/ Cited 5 Jan. 2018

[CR2] Akimoto K, Sano F, Homma T, Tokushige K, Nagashima M, Tomoda T (2014). Assessment of the emission reduction target of halving CO_2_ emissions by 2050: macro-factors analysis and model analysis under newly developed socio-economic scenarios. Energy Strategy Rev.

[CR3] BankTrack.org (2017) Deutsche Bank puts first restrictions on coal finance, but still has long way to go https://wwwbanktrackorg/news/deutsche_bank_puts_first_restrictions_on_coal_finance_but_still_has_long_way_to_go Cited 5 Sept. 2017

[CR4] Bertram C, Luderer G, Pietzcker RC, Schmid E, Kriegler E, Edenhofer O (2015). Complementing carbon prices with technology policies to keep climate targets within reach. Nat Clim Chang.

[CR5] Bertram C, Johnson N, Luderer G, Riahi K, Isaac M, Eom J (2015). Carbon lock-in through capital stock inertia associated with weak near-term climate policies. Technol Forecast Soc Change.

[CR6] Bohringer C, Rutherford T (2013). Transition towards a low carbon economy: a computable general equilibrium analysis for Poland. Energy Policy.

[CR7] BP (2015) Statistical review of world energy 2015. http://www.bp.com/en/global/corporate/energy-economics/statistical-review-of-world-energy.html. Cited 26 Jan. 2017

[CR8] Bryant G (2016). Creating a level playing field? The concentration and centralisation of emissions in the European Union Emissions Trading System. Energy Policy.

[CR9] Buckley T (2017) IEEFA update: global energy-finance transition gains steam. Available: http://ieefaorg/ieefa-update-global-energy-finance-transition-gains-steam/ Cited 8 Sept. 2017

[CR10] Bukowski M et al (2016) Revenues from ETS auctioning as source of financing for low-emission modernization in Poland. Forum for Energy Analysis. Available: wwwforum-energiieu/files/file_add/file_add-40pdf Cited 27 Jan. 2017

[CR11] Cabinet of Ministers (2014) Regulation of 8 April 2014 on the list of installations generating electricity, covered by the greenhouse gas emission allowance trading scheme, Journal of Laws 2014, item 472. Warsaw, Poland

[CR12] CAN – Climate Action Network Europe (2014) Stronger together investment support and solidarity mechanisms under the EU’s 2030 climate and energy framework. Report CAN Europe, Greenpeace and WWF, Sept 2014 (available at http://wwwgreenpeaceorg/eu-unit/Global/eu-unit/reports-briefings/2014/20140908%20Stronger%20Together%20CAN %20WWF%20Greenpeacepdf Cited 7 Sept. 2017

[CR13] Capros P (2006) Impacts of the EU ETS on electricity prices. Available http://14710223135/e3mlab/presentations/Impacts_of_the_EU_ETSpdf Cited 8 Sept. 2017

[CR14] Capros P, Paroussos L, Fragkos P, Tsani S, Boitier B, Wagner F, Busch S, Resch G, Blesl M, Bollen J (2014). European decarbonisation pathways under alternative technological and policy choices: a multi-model analysis. Energy Strategy Rev.

[CR15] Carbon-pulse.com. (2015) Portal http://carbon-pulse.com. Cited 9 Oct 2015

[CR16] CEE (2016) Bankwatch Network, Carbon Market Watch. Fossil fuel subsidies from Europe’s carbon market. The lessons learnt with article 10c of the EU ETS Directive and recommendations for the post-2020 period http://carbonmarketwatchorg/wp-content/uploads/2016/04/Fossil-fuel-subsidies-from-Europes-carbon-market-final web.pdf Cited 27 Jan. 2017

[CR17] Climate Analytics (2017). A stress test for coal in Europe under the Paris Agreement. Available: wwwclimateanalyticsorg/publications Cited 8 Sept. 2017

[CR18] Climate Scorecard (2016) Poland emission reduction challenges. Posted on 30 Aug 2016. http://climatescorecard.org/2016/08/30/poland-emission-reduction-challenges/. Cited 27 Jan. 2017

[CR19] DB – Deutsche Bank (2017) Amended guidelines for coal financing. https://www.db.com/newsroom_news/2017/medien/amended-guidelines-for-coal-financing-en-11466.htm. Cited 8 Sept. 2017

[CR20] Directive EED (2012) – Directive of the European Parliament and of the Council of 25 October 2012 on energy efficiency, amending directives 2009/125/EC and 2010/30/EU and repealing directives 2004/8/EC and 2006/32/EC

[CR21] Directive EU ETS (2009) – Directive 2009/29/EC of the European Parliament and of the Council of 23 April 2009 amending directive 2003/87/EC so as to improve and extend the greenhouse gas emission allowance trading scheme of the community

[CR22] Directive IED (2010) – Directive 2010/75/EU of the European Parliament and of the Council of 24 of November 2010 on industrial emissions

[CR23] Directive NEC (2016) – Directive 2016/2284 of the European Parliament and of the council of 14 December 2016 on the reduction of national emissions of certain atmospheric pollutants, amending directive 2003/35/EC and repealing Directive 2001/81/EC

[CR24] E3MLab & IIASA (2016) Technical report on Member State results of the EUCO policy scenarios. Available: https://eceuropaeu/energy/sites/ener/files/documents/20170125_-_technical_report_on_euco_scenarios_primes_correctedpdf Cited 5 Sept. 2017

[CR25] EC – European Commission (2011) Energy roadmap 2050, COM(2011) 885. https://ec.europa.eu/energy/sites/ener/files/documents/sec_2011_1565_part2pdf Cited 17 Jan. 2018

[CR26] EC – European Commission (2013) EU Energy, Transport and GHG Emissions Trends to 2050 Reference Scenario 2013. http://ec.europa.eu/transport/sites/transport/files/media/publications/doc/trends-to-2050-update-2013.pdf. Cited 27 Jan. 2017

[CR27] EC – European Commission (2015a) Report from the Commission to the European Parliament and the Council. Climate action progress report, including the report on the functioning of the European carbon market and the report on the review of Directive 2009/31/EC on the geological storage of carbon dioxide. COM(2015) 576 final

[CR28] EC – European Commission (2015b) Evaluation of the EU ETS Directive https://ec.europa.eu/clima/sites/clima/files/ets/revision/docs/review_of_eu_ets_en.pdf Cited 8 Sept. 2017

[CR29] EC – European Commission (2015c) Proposal for a Directive of the European Parliament and of the Council amending Directive 2003/87/EC to enhance cost-effective emission reductions and low-carbon investments 2015/148 (COD)

[CR30] EC – European Commission (2016a) Winter Package. https://eceuropaeu/priorities/priorities/energy-union-and-climate/proposals-clean-energy-all-europeans_en. Cited 27 Jan. 2017

[CR31] EC – European Commission (2016b) Proposal for a Regulation of the European Parliament and of the Council on the internal market for electricity. Brussels, 30112016 COM(2016) 861 final http://ec.europa.eu/energy/sites/ener/files/documents/1_en_act_part1_v9.pdf. Cited 27 Jan. 2017

[CR32] EC – European Commission (2016c) Clean energy for all Europeans, COM(2016) 860 final https://eceuropaeu/transparency/regdoc/rep/1/2016/en/com-2016-860-f1-en-main.pdf. Cited 8 Sept. 2017

[CR33] EC – European Commission (2016d) Commission staff working document accompanying the document energy prices and costs in Europe, SWD(2016) 420 final https://ec.europa.eu/energy/en/data-analysis/market-analysis. Cited 10 Sept. 2017

[CR34] EC – European Commission (2016e) Energy prices and costs in Europe COM(2016) 769 final https://ec.europa.eu/energy/en/data-analysis/market-analysis. Cited 8 Sept. 2017

[CR35] EC – European Commission (2016f) Governance of the Energy Union https://ec.europa.eu/energy/en/topics/energy-strategy-and-energy-union/governance-energy-union. Cited 10 Sept. 2017

[CR36] EC – European Commission (2016g) G20 Energy Ministers commit to tackle together global energy and climate challenges https://ec.europa.eu/energy/en/news/g20-energy-ministers-commit-tackle-together-global-energy-and-climate-challenges. Cited 18 Jan. 2018

[CR37] EC – European Commission (2017) EU ETS Revision for phase 4 (2021-2030), EC https://ec.europa.eu/clima/policies/ets/revision_en. Cited 14 Sept. 2017

[CR38] EEA – European Environment Agency (2015) Main themes and sectors addressed in the national State of Environment report http://www.eea.europa.eu/soer-2015/countries/poland. Cited 27 Jan. 2017

[CR39] EEA – European Environment Agency (2016a) European Environment Agency, Trends and projections in the EU ETS in 2016, EEA Report no. 24/2016. http://www.eea.europa.eu/publications/trends-and-projections-EU-ETS-2016. Cited 26 Jan. 2017

[CR40] EEA – European Environment Agency (2016b) Renewable energy in Europe 2016. EEA Report no. 4/2016. ISSN 1977-8449

[CR41] EEA – European Environment Agency (2016c) Transforming the EU power sector: avoiding a carbon lock-in, EEA Report no. 22/2016. ISBN: 978-92-9213-809-7 https://www.eea.europa.eu/publications/transforming-the-eu-power-sector Cited 18 Jan. 2018

[CR42] Endcoal.org (2016) Global coal plant tracker, Summary statistics. http://endcoal.org/global-coal-plant-tracker/summary-statistics/. Cited 27 Jan. 2017

[CR43] Energy Forum (2016) Challenges for the Polish power industry—recommendations http://forum-energii.eu/files/file/FAE_Polish_energy_in_Europe_Recommendations_27_01_2015.pdf. Cited 8 Sept. 2017

[CR44] Energy Post (2013) Report: Poland can handle higher carbon prices. July 11, 2013. http://energypost.eu/report-poland-can-handle-higher-carbon-prices/. Cited 26 Jan. 2017

[CR45] Eom J, Edmonds J, Krey V, Johnson N, Longden T, Luderer G, Riahi K, van Vuuren DP (2015). The impact of near-term climate policy choices on technology and emission transition pathways. Technolog Forecast Soc Chang.

[CR46] EPA – US Environmental Protection Agency (2017) Coal-Fired Power Plants Clean Up Their Act, https://www.epa.gov/Energy-Independence. Cited 8 Sept. 2017

[CR47] EPS Project (2017) Energy policy simulator report by the Polish National Energy Conservation Agency and the Energy Innovation: policy and technology LLC, Warsaw, 2017 https://energypolicy.solutions/docs/architectural-designhtml Cited 8 Sept. 2017

[CR48] Erickson P, Kartha S, Lazarus M, Tempest K (2015). Assessing carbon lock-in. Environ Res Lett.

[CR49] EUobserver (2017) US prevents G7 energy statement https://euobserver.com/energy/137558. Cited 29 Sept.2017

[CR50] EURACOAL (2017) Position paper on the “Clean energy for all Europeans” package https://euracoal.eu/library/position-papers/. Cited 8 Sept. 2017

[CR51] EurActiv (2014) Poland’s carbon emissions billions to be spent on coal, cutting budget deficit. 18.09.2014. Available: http://www.euractiv.com/section/energy/news/poland-s-carbon-emissions-billions-to-be-spent-on-coal-cutting-budget-deficit/.Cited 27 Jan. 2017

[CR52] EurActiv (2016) Lawmakers eye excluding coal from EU energy transition funds 25.11.2016. Available: http://www.euractiv.com/section/energy/news/lawmakers-eye-excluding-coal-from-eu-energy-transition-funds/. Cited 27 Jan. 2017

[CR53] EurActiv (2017) EU pollutant limits threaten large-scale coal plants, says new report Available:https://www.euractiv.com/section/air-pollution/news/eu-pollutant-limits-threaten-large-scale-coal-plants-says-new-report/. Cited 8 Sept. 2017

[CR54] Eurelectric (2016) EU ETS Reform—EURELECTRIC recommendations on proposals to strengthen the EU ETS. Eurelectric position paper. http://www.eurelectric.org/media/295167/20161130_recommendation_to_strengthen_eu_ets-2016-030-0608-01-e.pdf. Cited 27 Jan. 2017

[CR55] Eurelectric (2017) Impact assessment of a 550 Emission Performance Standard in capacity mechanisms. http://wwweurelectricorg/publications/filtered?pa=11693. Cited 15 October 2017

[CR56] Eur-Lex (2014) A policy framework for climate and energy in the period from 2020 to 2030

[CR57] European Parliament (2017) Post-2020 reform of the EU Emissions Trading System, http://www.europarl.europa.eu/thinktank/en/document.html?reference=EPRS_BRI(2017)599398. Cited 9 Sept. 2017

[CR58] Flisowska J (2017) Reformed carbon market must no longer fund coal. EurActiv Available: http://www.euractiv.com/section/emissions-trading-scheme/opinion/reformed-carbon-market-must-no-longer-fund-coal/. Cited 8 Sept. 2017

[CR59] Gawlik L, Mokrzycki E, Tiess G (2016). Poland: energy policy. Encyclopedia of mineral and energy policy.

[CR60] Goodman J, Marshall JP, Pearse R (2016). Coal, climate and development: comparative perspectives. Energy Policy.

[CR61] HEAL – Health and Environment Alliance (2017) Hidden price tags. How ending fossil fuels subsidies would benefit our health. Available online at: http://env-health.org/IMG/pdf/healthandenvironmentalliance_hidden_price_tags_report.pdf. Cited 15 Jan. 2018

[CR62] Healy N, Barry J (2017). Politicizing energy justice and energy system transitions: fossil fuel divestment and a “just transition”. Energy Policy.

[CR63] Healy S, Schumacher K, Stroia A, Slingerland S (2015) Review of literature on EU ETS Performance. A literature review and gap analysis of policy evaluations. Öko-Institut Working Paper 2 https://www.oeko.de/oekodoc/2455/2015-001-en.pdf. Cited 6 Sept. 2017

[CR64] Hoffmann V (2007). EU ETS and investment decisions: the case of the German electricity industry. Eur Manag J.

[CR65] IEA – International Energy Agency (2015a). IEA statistics. Available online at: iea.org/stats/index.asp. Cited 31 October 2016

[CR66] IEA – International Energy Agency (2015b). Report. Available: https://www.iea.org/statistics/statisticssearch/report/?country=POLAND&product=balances&year=2014. Cited 14 Sept. 2017

[CR67] IEA – International Energy Agency (2016a) Energy policies of IEA countries. Poland, 2016, https://www.iea.org/publications/freepublications/publication/energy-policies-of-iea-countries---poland-2016-review.html. Cited 8 Sept. 2017

[CR68] IEA – International Energy Agency (2016b) Medium-term coal market report 2016, https://www.iea.org/newsroom/news/2016/december/medium-term-coal-market-report-2016.html. Cited 2 Sept. 2017

[CR69] IEA – International Energy Agency (2017) World Energy Investment 2017 http://www.iea.org/publications/wei2017/. Cited 3 Sept. 2017

[CR70] IEE – The Institute of Energy Economics, Japan (2017) Energizing India. A Joint Project Report of NITI Aayog and IEEJ http://niti.gov.in/writereaddata/files/document_publication/Energy%20Booklet.pdf Cited 8 Sept. 2017

[CR71] IEEFA – Institute for Energy Economics and Financial Analysis (2016a) Brand-new Dutch coal plants are crashing in value. http://ieefa.org/16278-2/. Cited 2 Sept. 2017

[CR72] IEEFA – Institute for Energy Economics and Financial Analysis (2016b) The beginning of the end: fundamental changes in energy markets are undermining the financial viability of coal-fired power plants in Texas. http://ieefa.org/ieefa-texas-beginning-end-coal-fired-electricity-%E2%80%A8/. Cited 8 Sept. 2017

[CR73] IEEFA – Institute for Energy Economics and Financial Analysis (2017a) IEEFA update: signs in Bangladesh of a budding electricity-sector transition. http://ieefa.org/ieefa-update-signs-bangladesh-budding-electricity-sector-transition/. Cited 8 Sept. 2017

[CR74] IEEFA – Institute for Energy Economics and Financial Analysis (2017b) On the blogs: India turns purposefully away from coal. http://ieefa.org/blogs-india-turn-purposefully-away-coal/. Cited 9 Sept. 2017

[CR75] IGSMiE PAN – Mineral and Energy Economy Research Institute (2013) Forecast of demand for the Polish economy for hard coal and lignite as a raw material for the power sector in the 2050 perspective, Polish Academy of Sciences Report

[CR76] IPCC – Intergovernmental Panel on Climate Change (2006) Guidelines for National Greenhouse Gas Inventories. IPCC Publications vol2 Energy http://www.ipcc-nggip.iges.or.jp/public/2006gl/vol2.html. Cited 8 Sept. 2017

[CR77] IPCC – Intergovernmental Panel on Climate Change (2014) Summary for policymakers, Synthesis Report http://ar5-syr.ipcc.ch/topic_summary.php. Cited 6 Sept. 2017

[CR78] Kamiński J (2009). The impact of liberalisation of the electricity market on the hard coal mining sector in Poland. Energy Policy.

[CR79] Kamiński J (2011). Market power in a coal-based power generation sector: the case of Poland. Energy.

[CR80] Kamiński J, Kudełko M (2010). The prospects for hard coal as a fuel for the polish power sector. Energy Policy.

[CR81] Kavouridisa K, Koukouzas N (2008). Coal and sustainable energy supply challenges and barriers. Energy Policy.

[CR82] KOBIZE – National Centre for Emission Management at the Institute of Environmental Protection (2015) Poland’s National Inventory Report 2015. Greenhouse Gas Inventory for 1988–2013. Warsaw, October 2015

[CR83] Koch N, Fuss S, Grosjean G, Edenhofer O (2014). Causes of the EU ETS price drop: recession, CDM, renewable policies or a bit of everything?—new evidence. Energy Policy.

[CR84] Kowalski K (2016) In Poland, efforts to rescue coal industry will likely come up short. Midwest Energy News. Available: http://midwestenergynews.com/2016/07/12/in-poland-efforts-to-rescue-coal-industry-will-likely-come-up-short/. Cited 8 Sept. 2017

[CR85] Krabbe O et al (2016) Impact of EU ETS phase IV proposals on administrative costs and quality of the data collection process. ECOFYS. http://www.ecofys.com/files/files/impact-eu-ets-phase-iv-proposals-on-admin-costs-final.pdf. Cited 2 Sept. 2017

[CR86] Laing T et al (2014) The effects and side-effects of the EU emissions trading scheme. Wiley Interdisciplinary Reviews: Climate Change. ISSN 1757-7780 http://eprints.lse.ac.uk/56790/. Cited 2 Sept. 2017

[CR87] Lecuyer O et al (2014) Optimal transition from coal to gas and renewable power under capacity constraints and adjustment costs. Policy Research Working Paper no. 6985. http://hdl.handle.net/10986/19388. Cited 2 Sept. 2017

[CR88] Linares P (2006). Impacts of the European emissions trading scheme and permit assignment methods on the Spanish electricity sector. Energy.

[CR89] Lise W, Sijm J, Hobbs BF (2010). The impact of the EU ETS on prices, profits and emissions in the power sector: simulation results with the COMPETES EU20 model. Environ Resour Econ.

[CR90] McKinsey&Company (2009) Assessment of greenhouse gas emissions abatement potential in Poland by 2030. https://www.mckinsey.com/~/media/McKinsey/Business%20Functions/Sustainability%20and%20Resource%20Productivity/Our%20Insights/Greenhouse%20gas%20abatement%20potential%20in%20Poland/Assessment%20of%20greenhouse%20gas%20emissions%20abatement%20potential%20in%20Poland.ashx. Cited 8 Sept. 2017

[CR91] Ministry of Economy (2009) Energy policy of Poland till 2030. Appendix to Resolution no. 202/2009 of the Council of Ministers of 10 November 2009 http://climateobserver.org/wp-content/uploads/2014/09/Poland_EPP-2030-2009.pdf. Cited 26 Jan. 2017

[CR92] Ministry of Economy (2015) Poland 2015 Report Economy. Available: https://www.mr.gov.pl/media/15347/Poland_2015_Report_economy_eng.pdf. Cited 26 Jan. 2017

[CR93] Ministry of Energy (2015) Energy policy of Poland till 2050. BIP. October 2015. http://bip.me.gov.pl/node/24670. Cited 27 Jan. 2017

[CR94] Morgan S (2017) Finland doubles down on nuclear power as coal heads out the door. EurActiv https://www.euractiv.com/section/energy/news/finland-doubles-down-on-nuclear-power-as-coal-heads-out-the-door/. Cited 8 Sept. 2017

[CR95] Nachmany M et al (2015) Climate Change Legislation in Poland. An excerpt from the 2015 Global Climate Legislation Study. http://www.lse.ac.uk/GranthamInstitute/legislation/countries/poland/. Cited 26 Jan. 2017

[CR96] Nelson A (2017a) EU must shut all coal plants by 2030 to meet Paris climate pledges, study says. The Guardian, on-line, 9 Feb. 2017

[CR97] Nelson A. (2017b) The end of coal: EU energy companies pledge no new plants from 2020, The Guardian, on line, 5 Apr. 2017

[CR98] Neuhoff K et al (2006). Allocation, incentives and distortions: the impact of EU ETS emissions allowance allocations to the electricity sector 10.17863/CAM.5165. Cited 2 Sept. 2017

[CR99] NIK (2016) Adapting Polish industry to the requirements of the Energy and Climate Package: report of the Polish Supreme Audit Office https://www.nik.gov.pl/plik/id,12276,v,artykul_13584.pdf. Cited 27 Jan. 2017

[CR100] NIK (2017) Functioning of hard coal mining in the years 2007-2015 against the background of the government program: report of the Polish Supreme Audit Office. https://www.nik.gov.pl/aktualnosci/nik-o-gornictwie-wegla-kamiennego-w-latach-2007-2015.html. Cited 8 Sept. 2017

[CR101] Norges Bank (2017) Investment Management, Observation and exclusion of companies. https://www.nbim.no/en/responsibility/exclusion-of-companies/. Cited 8 Sept. 2017

[CR102] OECD/IEA (2014) The impact of global coal supply on worldwide electricity prices. http://www.iea.org/publications/insights/insightpublications/the-impact-of-global-coal-supply-on-worldwide-electricity-prices.html. Cited 8 Sept. 2017

[CR103] OECD/IEA (2015) Track the energy transition, COP 21. http://www.iea.org/publications/freepublications/publication/COP21EnergyTransition_DataBrief_08December.pdf. Cited 12 Sept. 2017

[CR104] O’Rourke-Potocki H (2016) Poland’s coal economy is in shambles, 1/4/16. Available: http://www.politico.eu/article/poland-duda-szydlo-coal-emissions-pollution-cop21/. Cited 7 Sept. 2017

[CR105] Polish TSO – Transmission System Operator (2016) website: the PSE S.A. (in Polish) http://www.pse.pl/index.php?dzid=115&did=581. Cited 31 Aug. 2017

[CR106] Reuters (2017) EXCLUSIVE-Polish “bluff” in EU climate talks tests bloc's patience. Reuters, 5 February 2017 http://news.trust.org/item/20170205080535-dd34d. Cited 8 Sept. 2017

[CR107] Reuters Staff (2017) Poland’s Enea to spend up to 500 mln zlotys to meet EU coal plant rules, Reuters, May 25, 2017 http://af.reuters.com/article/africaTech/idAFL8N1IR381. Cited 3 Sept. 2017

[CR108] Rogge Karoline S., Hoffmann Volker H. (2010). The impact of the EU ETS on the sectoral innovation system for power generation technologies – Findings for Germany. Energy Policy.

[CR109] RWE (2015) Technology scenarios for the polish energy market through 2050. RWE Study https://www.rwe.com/web/cms/mediablob/de/2560854/data/184336/4/rwe/innovation/Technology-scenarios-for-the-polish-energy-market-through-2050.pdf. Cited 26 Aug. 2017

[CR110] Sanzillo T (2016) Notes on the EPA’s Clean Power Plan (Part 4: why America should reinvest in coal communities). IEEFA, http://ieefa.org/notes-on-the-epas-clean-power-plan-part-4-why-america-should-reinvest-in-coal-communities/. Cited 8 Sept. 2017

[CR111] Skoczkowski T, Wronka A (2017). Analysis of EU ETS reforms from Poland’s power sector perspective. Przegląd Elektrotechniczny R.93 3/2017:212–222

[CR112] SourceWatch (2017) Coal plant retirements http://www.sourcewatch.org/index.php/Coal_plant_retirements. Cited 8 Sept. 2017

[CR113] Stavis R (2017) Why Trump pulled the U.S. out of the Paris accord and what the consequences will be. In Foreign Affairs http://www.robertstavinsblog.org/2017/06/05/trump-pulled-u-s-paris-accord/. Cited 8 Sept. 2017

[CR114] Stokka O (2015) Output-based allocation of emission permits in the EU ETS. NMBU. Available: https://www.nmbu.no/download/file/fid/17129. Cited 8 Sept. 2017

[CR115] Suwała W (2008). Modelling adaptation of the coal industry to sustainability conditions. Energy.

[CR116] Suwała W, Labys W (2002). Market transition and regional adjustments in the Polish coal industry. Energy Econ.

[CR117] TOE (2016) The Association of Energy Trading Electricity and Gas Market in Poland. Status on March 31, 2016. TOE Report. http://www.toe.pl/pl/wybrane-dokumenty/rok-2016?download=1462:electricity-and-gas-market-in-poland-status-on-31-march-2016-toe-report Cited 27 Jan. 2017

[CR118] URE (2017) Energy Regulatory Office in Poland. Report on the activities of the President of the Energy Regulatory Office in Poland in 2016 https://www.ure.gov.pl/pl/urzad/informacje-ogolne/sprawozdania/2916,Sprawozdania.html. Cited 8 Sept. 2017

[CR119] Vallés M et al (2012) Impact of the EU ETS on the European electricity sector. 9th International Conference on the European Energy Market. doi:2012.6254801

[CR120] WEC – World Energy Council (2016) Efficiency of thermal power plants. Available: https://www.wec-indicators.enerdata.eu/industrial-chp.html#/power-plants-thermals.html. Cited 4 Nov. 2016

[CR121] Weishaar S (2016) Research handbook on emissions trading. Edward Elgar Publishing. 10.4337/9781784710620

[CR122] Wierzbowski M, Filipiak I, Lyzwa W (2017). Polish energy policy 2050—an instrument to develop a diversified and sustainable electricity generation mix in coal-based energy system. Renew Sust Energ Rev.

[CR123] Włodarczak M (2016) An assessment of the impact of EU ETS on the Polish conventional power sector over the period 2008–2020. Master Thesis, Warsaw University of Technology, Faculty of Power and Aeronautical Engineering

[CR124] wnp.pl (2016) Power market in Poland—who would be excluded by the criterion 550 kg CO2/MWh?: http://energetyka.wnp.pl/rynek-mocy-w-polsce-kogo-wykluczyloby-kryterium-550-kg-co2-mwh,288138_1_0_0.html. Cited 27 Jan. 2017

[CR125] Wood I, Broom R (2016). Reforming Poland’s renewable industry. Renewable Energy Focus.

[CR126] World Bank (2011) Transition to a low carbon economy in Poland. Energy Sector Management Assistance Program (ESMAP). http://hdl.handle.net/10986/17495. Cited 8 Sept. 2017

[CR127] World Bank (2013) Toward a sustainable energy future for all: directions for the World Bank Group’s energy sector. http://documents.worldbank.org/curated/en/745601468160524040/Toward-a-sustainable-energy-future-for-all-directions-for-the-World-Bank-Group-8217-s-energy-sector. Cited 8 Sept. 2017

[CR128] World Bank (2014a) World Bank https://data.worldbank.org/indicator/EN.ATM.CO2E.KD.GD?locations=PL-EU Cited 1 Sept. 2017

[CR129] World Bank (2014b). Poland - Energy Efficiency Development Policy Loan (English). Washington, DC: World Bank Group. http://documents.worldbank.org/curated/en/211791474586622602/Poland-Energy-Efficiency-Development-Policy-Loan. Cited 10 Sept. 2017

[CR130] World Bank (2015) Romania toward a low carbon and climate resilient economy: energy sector analysis. http://hdl.handle.net/10986/24060. Cited 2 Sept. 2017

[CR131] World Bank (2016) Carbon pricing watch 2016. http://www.ecofys.com/files/files/world-bank-group_ecofys-carbon-pricing-watch_160525.pdf. Cited 8 Sept. 2017

[CR132] World Bank (2017a) Poland’s success is remarkable, but further reforms are needed, press release. http://www.worldbank.org/en/news/press-release/2017/03/21/poland-success-is-remarkable-but-further-reforms-are-needed. Cited 2 Sept. 2017

[CR133] World Bank (2017b) Report of the high-level commission on carbon prices, May 2017 https://www.researchgate.net/publication/318284315. Cited 2 Sept. 2017

[CR134] World Bank (2017c) Regulatory indicators for sustainable energy, a Global Scorecard for Policy Makers http://documents.worldbank.org/curated/en/538181487106403375/Regulatory-indicators-for-sustainable-energy-a-global-scorecard-for-policy-makers. Cited 2 Sept. 2017

[CR135] Global Tracking Framework 2017—Progress toward sustainable energy (World Bank 2017d)

[CR136] World Bank (2017d) Global Tracking Framework 2017—Progress toward sustainable energy http://www.worldbank.org/en/topic/energy/publication/global-tracking-framework-2017. Cited 18 Jan. 2018

[CR137] Wynn G, Coghe P (2017) Europe’s coal-fired power plants: rough times ahead. IEEFA. http://ieefa.org/wp-content/uploads/2017/05/Europe-Coal-Fired-Plants_Rough-Times-Ahead_May-2017.pdf. Cited 2 Sept. 2017

